# Polymer chimera of stapled oncolytic peptide coupled with anti-PD-L1 peptide boosts immunotherapy of colorectal cancer

**DOI:** 10.7150/thno.71129

**Published:** 2022-04-24

**Authors:** Lu Lu, He Zhang, Yudong Zhou, Jiayi Lin, Weidong Gao, Ting Yang, Jinmei Jin, Lijun Zhang, Dale G. Nagle, Weidong Zhang, Ye Wu, Hongzhuan Chen, Xin Luan

**Affiliations:** 1 Shanghai Frontiers Science Center for Chinese Medicine Chemical Biology, Institute of Interdisciplinary Integrative Medicine Research, Shanghai University of Traditional Chinese Medicine, Shanghai, 201203, China; 2 School of Pharmacy, Fudan University, Shanghai, 201203, China.; 3 Department of Chemistry and Biochemistry, College of Liberal Arts, University of Mississippi, University, MS, 38677-1848, USA; 4 Department of BioMolecular Sciences and Research Institute of Pharmaceutical Sciences, School of Pharmacy, University of Mississippi, University, MS 38677-1848, USA

**Keywords:** colorectal cancer, stapled mastoparan peptide, peptide-polymer conjugate, immunogenic cell death, oncolytic immunotherapy

## Abstract

**Rationale:** Scarce tumor mutation burden and neoantigens create tremendous obstacles for an effective immunotherapy of colorectal cancer (CRC). Oncolytic peptides rise as a promising therapeutic approach that boosts tumor-specific immune responses by inducing antigenic substances. However, the clinical application of oncolytic peptides has been hindered because of structural instability, proteolytic degradation, and undesired toxicity when administered systemically.

**Methods:** Based on wasp venom peptide, an optimized stapled oncolytic peptide MP9 was developed with rigid α-helix, protease-resistance, and CRC cell cytotoxicity. By incorporating four functional motifs that include D-peptidomimetic inhibitor of PD-L1, matrix metalloproteinase-2 (MMP-2) cleavable spacer, and MP9 with 4-arm PEG, a novel peptide-polymer conjugate (PEG-MP9-aPDL1) was obtained and identified as the most promising systemic delivery vehicle with PD-L1 targeting specificity and favorable pharmacokinetic properties.

**Results:** We demonstrated that PEG-MP9-aPDL1-driven oncolysis induces a panel of immunogenic cell death (ICD)-relevant damage-associated molecular patterns (DAMPs) both* in vitro* and *in vivo*, which are key elements for immunotherapy with PD-L1 inhibitor. Further, PEG-MP9-aPDL1 exhibited prominent immunotherapeutic efficacy in a CRC mouse model characterized by tumor infiltration of CD8+ T cells and induction of cytotoxic lymphocytes (CTLs) in the spleens.

**Conclusion:** Our findings suggest that PEG-MP9-aPDL1 is an all-in-one platform for oncolytic immunotherapy and immune checkpoint blockade (ICB).

## Introduction

Colorectal cancer (CRC) is the third most common high mortality malignancy 1. Although FOLFOX (combination of folinic acid, 5-fluorouracil, and oxaliplatin) is used as the first-line treatment for CRC 2, its use is associated with adverse effects and limited response rate, which is typical for conventional chemotherapy 3. Programmed cell death 1-ligand 1 (PD-L1)/programmed cell death protein-1 (PD-1)-targeted therapies represent new approaches that activate systemic antitumor immunity 4. However, only 5% of CRC patients are responsive to anti-PD-1/L1 agents and the response does not last in some patients 5. Clinical studies revealed that patients with deficient mismatch repair (dMMR) or microsatellite instable-high (MSI-H) tumors respond to immune checkpoint inhibition, in part because high mutation burden and subsequent neoantigen generation lead to the activation and infiltration of cytotoxic T cells 6, 7. Thus, it is not surprising that the majority of mismatch repair-proficient (pMMR) CRC patients do not respond to therapeutic immune checkpoint blockade 8.

Unlike immune checkpoint inhibitors, peptide-mediated oncolytic immunotherapy has been used to jumpstart the so-called cancer immunity cycle 9, specifically, the release of tumor-associated antigens (TAAs) to the tumor microenvironment (TME) led to rapid membrane disruption and immunogenic cell death (ICD) of cancer cells, thereby triggering a pronounced anti-tumor immune response 10. Oncolytic peptides afford the therapeutic advantage of combining tumor-specific cell lysis with immune stimulation, therefore acting as potential in situ tumor vaccines 11. The first-in-class oncolytic peptide LTX-315 is currently undergoing Phase II clinical trials. Following treatment, LTX-315 elicits oncolysis that releases a cohort of chemotactic and immune-stimulatory signals, turning neoplastic lesions with poor immunogenicity ('cold' tumors) into those with highly infiltration ('hot' tumors), amenable to enhanced anti-PD-1/L1 immunotherapy 12.

The intrinsic shortcomings of current oncolytic peptides include structural instability, proteolytic susceptibility, and suboptimal antitumor activity. To overcome these drawbacks, we developed a series of wasp venom-derived oncolytic peptides by peptide stapling technology, which significantly improved the α-helical stabilization and anti-tumor effects of parent peptide in B16F10 tumor-bearing mice 13. However, the requirement for intratumoral administration limits the application of these oncolytic peptides towards both large and metastatic tumors. Numerous studies have found that peptide-polymer conjugate 14, a hybrid macromolecule of peptides and polymer materials, can systematically deliver peptide drugs with the advantages of target specificity, reduced toxicity, and prolonged half-life in circulation 15. Therefore, it was hypothesized that biocompatible polymer could serve as a robust systemic delivery platform for stapled oncolytic peptides, which can induce tumor lysis and TAA release, and improve the efficacy of PD-L1 inhibitor-based immunotherapy for CRC.

In this study, a library of ten mastoparan-based stapled oncolytic peptides was synthesized, and the stable, potent, and selective MP9 was identified following structure-function-hemolysis studies. We also cleverly developed a versatile strategy for engineering prodrug peptide-polymer chimera, specifically, coupling D-type PD-L1 targeting peptide with 4-arm polyethylene glycol (PEG)-functionalized stapled peptide MP9 via a matrix metalloproteinase-2 (MMP-2)-sensitive linker.

As anticipated, the novel conjugate PEG-MP9-aPDL1 showed promising targeting and oncolytic efficacy *in vitro* and *in vivo*, most likely due to the high binding affinity for PD-L1 and long half-life. In MMP-2-rich TME, the anti-PD-L1 peptide can be cleaved from the prodrug PEG-MP9-aPDL1 that accumulated in tumors to impose immune checkpoint blockade, while exposing the stapled peptide MP9 to initiate oncolysis, followed by TAA release and ICD induction, which strengthened tumor infiltration of cytotoxic lymphocytes and resulted in enhanced oncolytic immunotherapy for CRC.

## Results

### Design, synthesis, and screening of stapled MP derivatives

**Figure 1:**
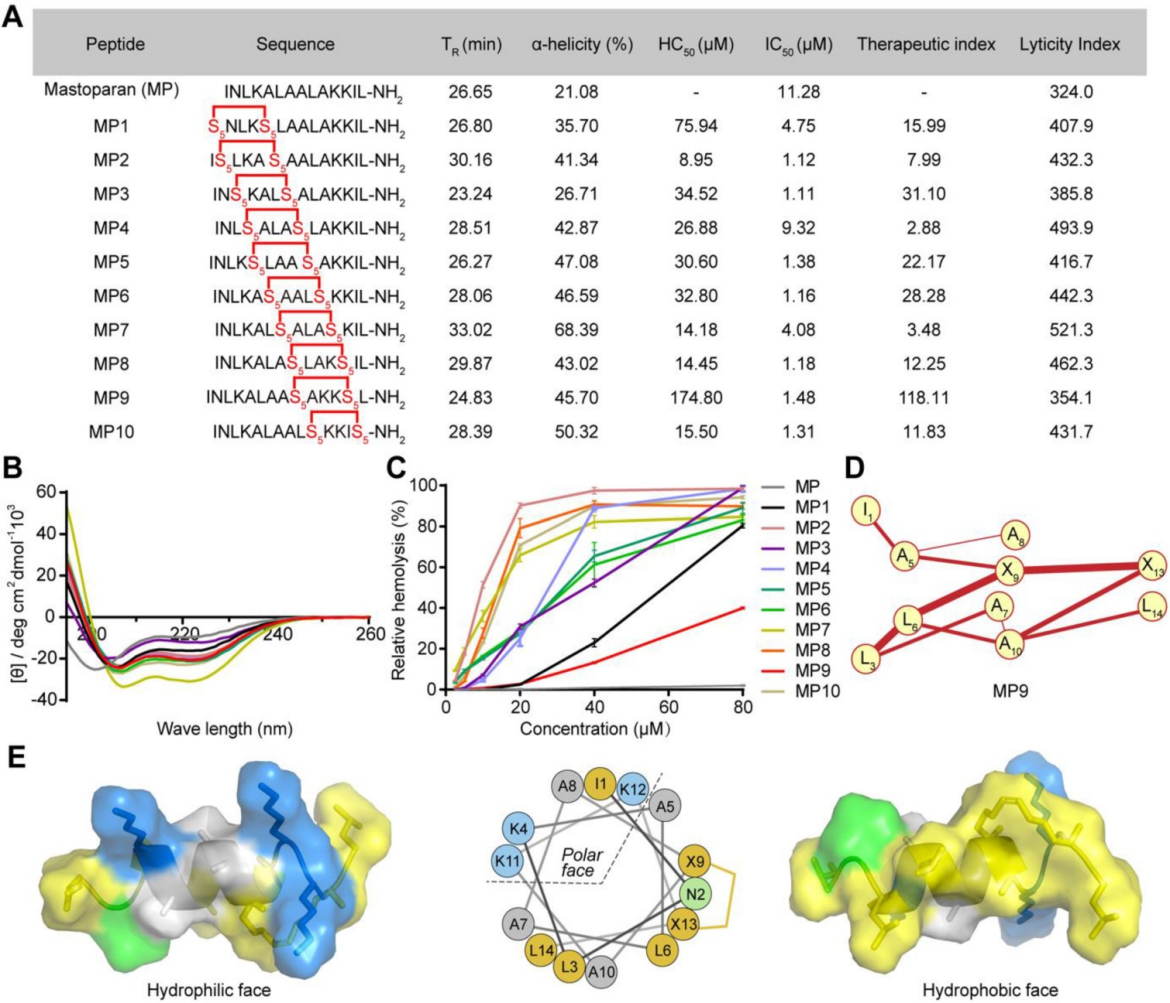
** α-helicity, antiproliferative and hemolytic activities of stapled MP derivatives. (A)** Sequence, C18-HPLC retention time (T_R_), α-helicity, hemolytic activity (HC_50_, half maximal hemolysis concentration), antitumor activity (IC_50_, half maximal inhibitory concentration), therapeutic index (HC_50_/IC_50_), and lyticity index of MP and derivatives. S_5_ = (*S*)-pentenyl alanine residue. **(B)** CD spectra of MP and derivatives. **(C)** Red blood cell (RBC) hemolytic activity of MP and derivatives (n = 3). **(D)** HNM of MP9. **(E)** Surface and cartoon views of the hydrophilic (left) and hydrophilic (right) faces of the amphipathic MP9 α-helix. The hydrophobic face contains two patches of highly hydrophobic residues (L, yellow) separated by low hydrophobic residues (A, gray) and polar, uncharged residue (N, green). Cationic residue (K) is represented in blue on the structures and helical wheel.

The antimicrobial peptide mastoparan (MP, INLKALAALAKKIL), isolated from the venom of the wasp *Vespula lewisii*, is an α-helix peptide with 14 amino acid residues 16. MP exhibited potent antitumor activity because of the interaction between its cationic amphipathic α-helical region and tumor cell membranes 17, 18. In order to enhance its oncolytic potency and resistance to enzyme degradation, an all-hydrocarbon stapling strategy was used to induce a stable α-helical structure 13. To identify MP derivative(s) with an optimal therapeutic index (TI), we incorporated two (*S*)-pentenyl alanine residues (S_5_) at the *i* and *i* + 4 positions of MP to obtain a staple-scanning library by Fmoc solid-phase peptide synthesis (SPPS) 19, 20 and evaluated their secondary structure features, anti-proliferative, and hemolytic activities (Figure 1A).

To confirm conformational changes induced by the all-hydrocarbon stapling strategy, the secondary structure of these peptides was determined by circular dichroism (CD). Analysis of the CD spectra indicated that the α-helicity of MP was only 21.08%, whereas the α-helical content value for MP1 to MP10 ranged from 35.70% to 68.39%, corresponding to a 1.69- to 3.24-fold increase (Figure 1A-B). These results suggested that conformational constraint could effectively stabilize the helical structure of MP, relative to its linear form. These stapled peptides were evaluated for cytotoxicity in the CRC cell line CT26 *in vitro*, IC_50_ values ranged from 1.11 μM to 9.32 μM. These stapled MP derivatives exhibited either enhanced or same anti-CRC activity, in comparison to the parent MP. Therefore, the MP stable α-helix conformation is preferable for enhancing anti-CRC activity (Figure 1A).

The potential of the stapled MP peptides to non-specifically lyse red blood cells (RBC) was measured and their hemolytic activities (HC_50_) determined. The peptide MP9 displayed the lowest level of RBC hemolysis (Figure 1A, C). According to the computational algorithm devised by the Loren group 21, we generated the lyticity index (LI) and “hydrophobicity network maps” (HNMs) of the peptides *in silico* (Figure 1A, S1), as indicators for the extent of nonspecific hemolysis and hydrophobic interactions on the α-helical surface, respectively. In consistence with the HC_50_ values, there was a positive correlation between the percentage of hemolysis and the calculated LI values. Among the samples examined, MP9 displayed the highest selectivity. Furthermore, MP9 preserved the structural characteristics of the linear MP (hydrophobic network, amphipathic α-helix, charge and solubility; Figure 1A, D-E), properties that are crucial for the balance between desired bioactivity and minimal hemolysis. Taken collectively, these data indicated that MP9 is the optimal candidate for further evaluation.

### Oncolytic activities study of stapled peptide MP9 *in vitro*

**Figure 2:**
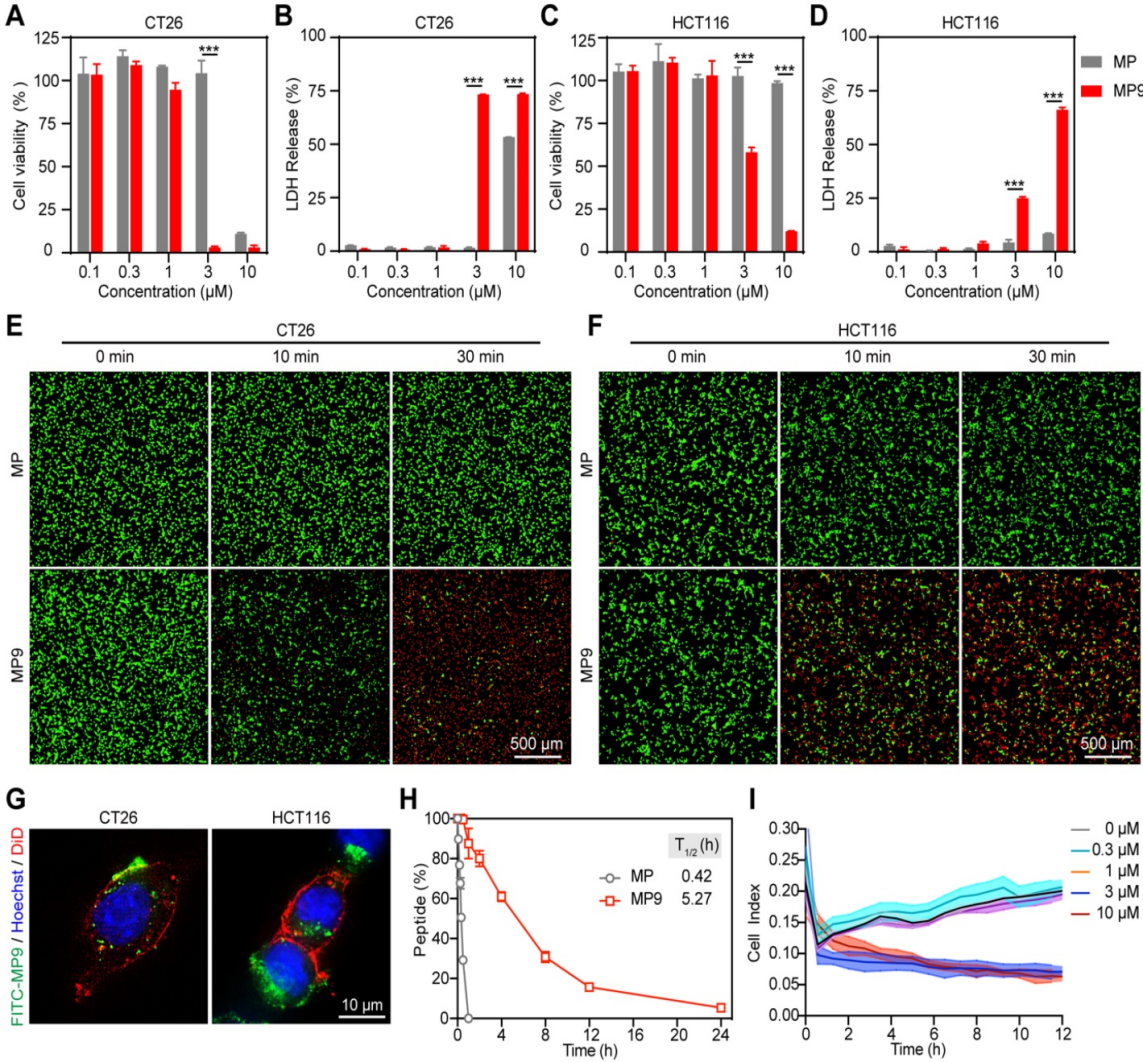
**MP9 exhibits potent and rapid oncolytic activity to CRC cells.** Cell viability analysis of CT26 **(A)** and HCT116 cells **(C)** cultured in the MP and MP9 for 24 h by CCK-8 assay. LDH release of CT26 **(B)** and HCT116 cells **(D)** following treatment with MP and MP9. CT26 **(E)** and HCT116 cells **(F)** after treatment with MP and MP9 and stained with the LIVE/DEAD cell viability/cytotoxicity kit. **(G)** The lytic effect of FITC-MP9 on CT26 and HCT116 cells, as observed by a confocal microscope. **(H)** Proteolytic stability of peptides MP and MP9 incubated in α-Chymotrypsin solution. **(I)** Kinetic curves of the cytotoxicity of CT26 cells after treatment with MP9 in different concentrations, as assessed by xCELLigence RTCA-DP. Data are presented as mean ± s.d.; n = 3. The representative photographs of the cells were shown. Statistical significance: ***p < 0.001.

Lactate dehydrogenase (LDH) release is often used as an indicator for the extent of cell lysis. Treatment of CRC cells CT26 and HCT116 with MP9 induced the release of LDH in a concentration-dependent manner (Figure 2B, D), which was consistent with the CCK-8 cell viability results (Figure 2A, C). To monitor MP9-induced tumor cell death, a calcein-AM and propidium iodide (PI) dual staining assay was used. Upon MP9 treatment, cell death was rapid and time-dependent (> 50% CT26 cells died within 30 minutes), and CT26 cells were more sensitive to MP9 than HCT116 cells (Figure 2E-F). In contrast, the parent MP peptide did not display significant cell lytic activity. Treatment with FITC-MP9 led to membrane disruption and lysis of CT26 and HCT116 cells (Figure 2G).

An α-chymotrypsin-mediated degradation assay was employed to determine proteolytic stability. After 1 h exposure, the parent MP peptide was completely degraded, while over 87% of MP9 remained intact, suggesting that the stapled peptide MP9 had greater protease stability with a 12.55-fold increase in half-life (T_1/2_) (Figure 2H). These results revealed that MP9 not only exhibited potent and rapid oncolytic activity by lysing tumor cell membrane, but also displayed a prolonged half-life.

An xCELLigence system, which measures cell proliferation by detecting electrical impedance via microelectronic biosensor technology, was used for Real-Time Cell Analysis (RTCA) to obtain quantitative cytotoxicity data. Following addition of MP9 to CT26 cells in culture, data were collected at 1 min intervals to record kinetic curves in real-time. At the concentrations of 3 and 10 μM, MP9 significantly reduced CT26 cell viability when compared to the untreated control, which supports the crucial role of MP9 interaction in suppressing CRC cells (Figure 2I).

### Design, synthesis, characterization of peptide-polymer conjugates

While oncolytic peptides have shown clinical potential for treating solid tumors, the requirement for intra-tumoral administration poses a huge challenge for developing oncolytic peptides as systemic treatment for distant or metastatic tumors 22. Because of the biocompatibility, improved pharmacokinetic properties and limited potential side effects, hydrophilic PEG has been approved for biomedical use by the FDA 23, 24. Thus, we chose PEG as a formulation for synthesizing peptide-polymer conjugate.

**Figure 3:**
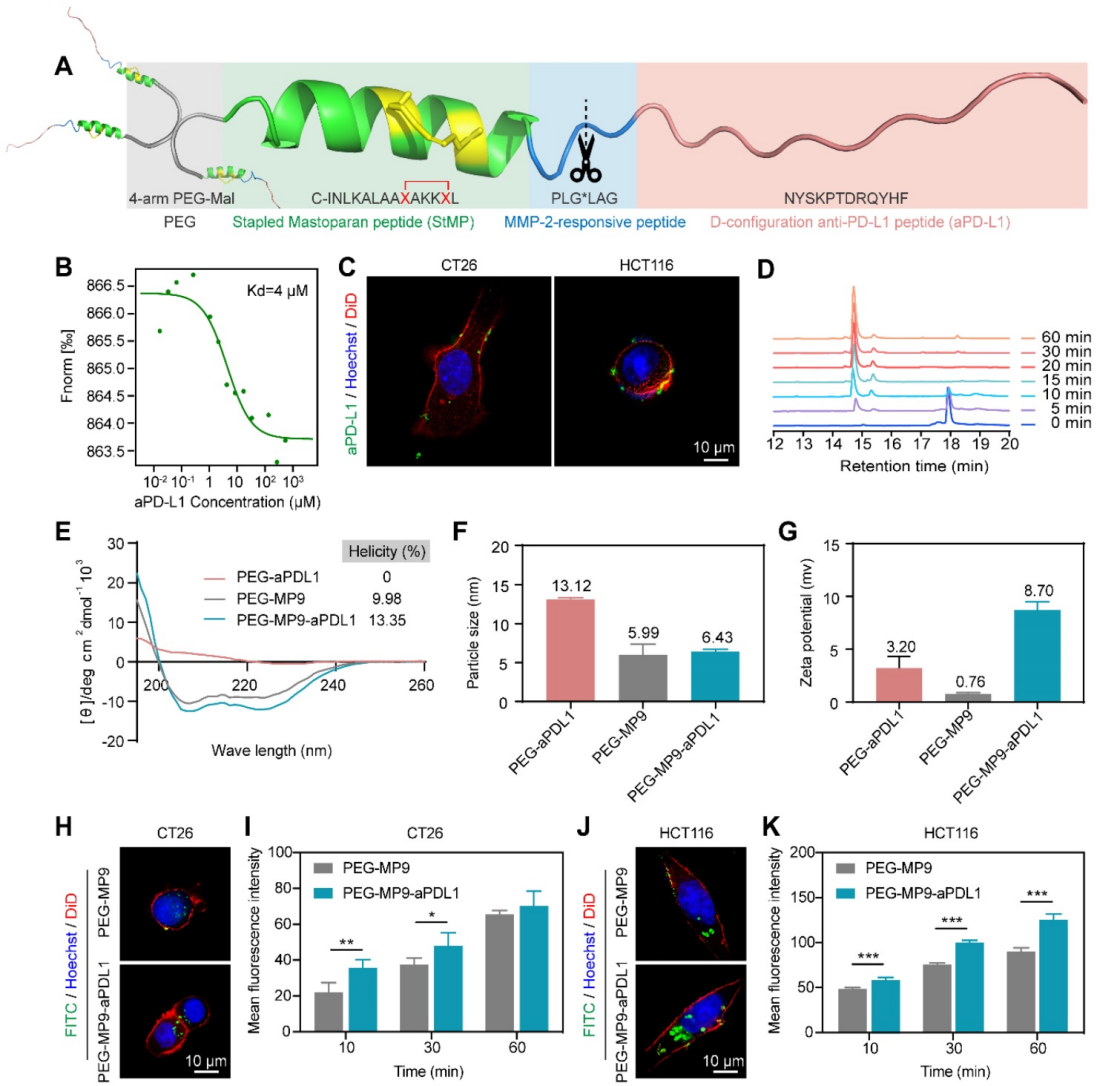
** Design, synthesis, and oncolytic effect of peptide-polymer conjugates. (A)** Schematic illustration of the construction of peptide-polymer conjugate PEG-MP9-aPDL1. **(B)** MST analysis of aPD-L1 and PD-L1 binding (*K_d_* = 4 μM). **(C)** Confocal microscopic images of the FITC-aPD-L1 peptide localized on CT26 and HCT116 cell membrane. **(D)** HPLC traces of peptide MMP-2-aPD-L1 treated with exogenous MMP-2 for different length of time. **(E)** CD spectra of PEG-aPDL1, PEG-MP9, and PEG-MP9-aPDL1. Particle size **(F)** and zeta potential **(G)** of PEG-aPDL1, PEG-MP9, and PEG-MP9-aPDL1 were measured by dynamic light scattering (DLS). Confocal microscopic images showed cellular uptake of FITC-PEG-MP9 and FITC-PEG-MP9-aPDL1 after 30 min treatment in CT26 **(H)** and HCT116 cells **(J)**. The mean fluorescence intensity (MFI) of CT26 **(I)** and HCT116 cells **(K)** after incubation with PEG-MP9 and PEG-MP9-aPDL1, respectively, for different amount of time. Data are presented as mean ± s.d.; n = 3. Representative photographs were shown. Statistical significance: *p < 0.05, **p < 0.01, and ***p < 0.001.

Due to the lack of *in vivo* selectivity 25, a targeting D-type anti-PD-L1 peptide (NYSKPTDRQYHF, aPD-L1) was incorporated into the MP9 conjugate (Figure 3A). This aPD-L1 peptide improves protease resistance and facilitates PD-L1 binding 26. A K_d_ value of 4 μM was observed between the aPD-L1 peptide and PD-L1 protein in MST experiment (Figure 3B), which is consistent with previous report 27. We also detected PD-L1 expression on primary human umbilical vascular endothelial cell (HUVEC) and CRC cell lines CT26 and HCT116. The level of PD-L1 expression on normal HUVECs was much lower than those of tumor cells (Figure S2). The MMP-2 sensitive linker (PLG*LAG) was introduced to connect the MP9 and aPD-L1 peptides, forming a bifunctional peptide (MP9-MMP-2-aPD-L1), which afforded both tumors targeting specificity and TME sensitivity (Figure 3A). Confocal microscopy images showed that the FITC labeled aPD-L1 peptide co-localized with CT26 and HCT116 cell membrane (Figure 3C). Analytical HPLC (214 nm) confirmed that the MMP-2-sensitive linker is cleavable by exogenous MMP-2 in a time-dependent manner (Figure 3D, S3).

The peptide-polymer chimera 4-arm PEG-MP9-MMP-2-aPD-L1 (PEG-MP9-aPDL1) was prepared by conjugating 4-arm PEG maleimide with four modified MP9-MMP-2-aPD-L1 peptides at the N-terminal of an additional Cys residue. This conjugate platform included four units (Figure 3A): the stapled peptide, the MMP-2-sensitive cleavable substrate peptide, the PD-L1 blocking aPD-L1 peptide, and a protective circulation-stable 4-arm PEG chain (Figure 3A). To obtain a peptide-polymer conjugate for systematic administration and targeted delivery, the hybrid peptide MP9-MMP-2-aPD-L1 was covalently coupled to the polymeric material. Once the hybrid peptide was cleaved in TME, not only could the free anti-PD-L1 peptides bind to PD-L1 on the surface of tumor cells to block tumor cells immune escape, but also allowed the exposed oncolytic peptide MP9 quickly kill tumor cells and release TAAs to induce ICD.

The peptide Cys-MP9 and peptide Cys-MMP-2-aPD-L1 were coupled with 4-arm PEG maleimide to obtain the polymer chimeras 4-arm PEG-MP9 (PEG-MP9) and 4-arm PEG-MMP-2-aPD-L1 (PEG-aPDL1) in the same way we used to prepare the controls. The conjugation efficiency of chimeras ranged from 85.15% to 92.02%, suggesting highly reaction efficiency (Table S2). ^1^H NMR studies revealed that these conjugates had an increase in the characteristic peaks of the polymer material PEG, when compared to free peptides (Figure S4-10). Analysis of the CD spectra suggested that the PEG-MP9-aPDL1 and PEG-MP9 conjugates maintained their α-helical MP9 structure characterized as double strong negative absorption peaks at 208 and 222 nm in the CD spectra, while PEG-aPDL1 presented negligible secondary structure (Figure 3E). Moreover, dynamic light scattering (DLS) (Figure 3F) measurements indicated that all the conjugates had the hydrodynamic size of approximately 10 nm. The cationic sequence of MP9 and aPD-L1 peptide conjugates exhibited positive ζ potential (0.76-8.70 mv) (Figure 3G) and were regular and rounded under transmission electron microscopy (TEM) (Figure S11). Interestingly, the size of PEG-MP9-aPDL1 was larger than PEG-aPDL1 and PEG-MP9 under TEM observation. However, the DLS size of PEG-aPDL1 was the biggest among all chimeras. We speculated that the stapled α-helix structure of MP9 restricted the stretching of aPD-L1 peptide in PEG-MP9-aPDL1 (Figure 3E).

In order to evaluate the intracellular uptake and localization of PEG-MP9 and PEG-MP9-aPDL1, fluorescent conjugates were prepared with FITC as the fluorophore. Following incubation with FITC-PEG-MP9 and FITC-PEG-MP9-aPDL1 for 30 min, super resolution microscopic studies were performed. Cells treated with FITC-PEG-MP9-aPDL1 exhibited a brighter green fluorescence in the cytoplasm of CT26 (Figure 3H-I) and HCT116 (Figure 3J-K) cells than those treated with FITC-PEG-MP9. This uptake could be completely inhibited following pre-incubation with free aPD-L1 peptide, suggesting that the improved targeting effect resulted from the specific interaction between aPD-L1 peptide and PD-L1 located on the surface of these two CRC cell lines (Figure S12). Using an ultimate live cell imaging microscope with the 3D Cell Explorer software, we observed the effects of free peptide MP9 and its chimera PEG-MP9-aPDL1 on CT26 and HCT116 cells in action. As shown in Figure S13, the adherent tumor cells lysed rapidly and released a large number of membrane fragments, within 10 min of treatment.

### PEG-MP9-aPDL1-mediated oncolysis induces immunogenic cell death in CRC cells

**Figure 4:**
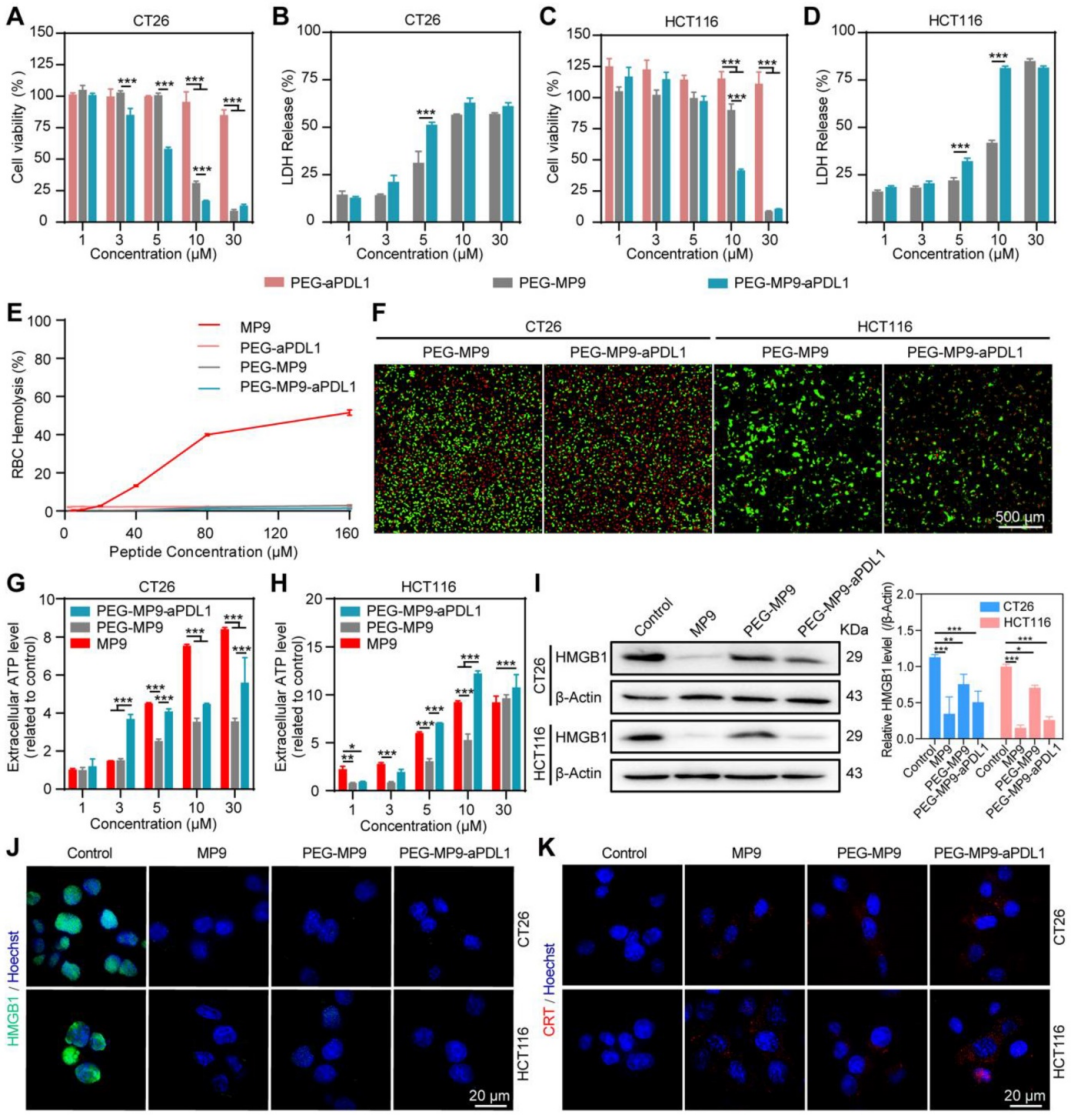
**PEG-MP9-aPDL1-mediated oncolysis induces immunogenic cell death of CRC cells.** Concentration-response results of CT26 **(A)** and HCT116 cells **(C)** cultured in the presence of PEG-MP9-aPDL1, PEG-MP9, and PEG-aPDL1 for 24 h and cell viability determined by CCK-8 assay. LDH release of CT26 **(B)** and HCT116 cells **(D)** following treatment with PEG-MP9-aPDL1, PEG-MP9, and PEG-aPDL1. **(E)** RBC hemolytic activity of MP9, PEG-aPDL1, PEG-MP9, and PEG-MP9-aPDL1. **(F)** CT26 and HCT116 cells after treatment with PEG-MP9-aPDL1, PEG-MP9, and PEG-aPDL1 and stained with the LIVE/DEAD cell viability/cytotoxicity kit. Extracellular ATP levels of CT26 **(G)** and HCT116 cells **(H)** following free MP9, PEG-MP9, and PEG-MP9-aPDL1 treatment. **(I)** Expression of HMGB1 protein in CT26 and HCT116 cells after treatment with MP9, PEG-MP9, and PEG-MP9-aPDL1 for 6 h, detected by Western blot. Confocal microscopic images showing HMGB1 **(J)** and CRT **(K)** in CT26 and HCT116 cells incubated with MP9, PEG-MP9, and PEG-MP9-aPDL1 for 6 h. Data are presented as mean ± s.d.; n = 3. Statistical significance: *p < 0.05, **p < 0.01, and ***p< 0.001.

Both the free stapled peptide MP9 and the PEGylated copolymer PEG-MP9-aPDL1 exhibited promising oncolytic potential. Cell viability studies revealed that the synthesized conjugate PEG-aPDL1 was not cytotoxic to CT26 (Figure 4A) and HCT116 (Figure 4C) cells at the concentration range of 1 to 30 μM. In contrast, PEG-MP9-aPDL1 caused considerable cytotoxicity to both CT26 and HCT116 cells with IC_50_ values of 6.41 μM and 13.33 μM, respectively. The IC_50_ values for PEG-MP9 were 13.51 μM and 23.14 μM in CT26 and HCT116, respectively. The antitumor activity of PEG-MP9-aPDL1 was decreased when pre-incubated with free anti-PD-L1 (Figure S14). These results indicated that PEG-MP9-aPDL1 was more potent than PEG-MP9 on account of improved targeting with aPD-L1. Similarly, exogenous MMP-2 also could generate decreased cytotoxicity of PEG-MP9-aPDL1, which suggested the successful cleavage of responsive linker and targeting ability of anti-PD-L1 peptide conjugation (Figure S15). By measuring LDH content in the cell culture supernatant, it was found that PEG-MP9-aPDL1 and PEG-MP9 disrupted the membrane integrity of two CRC cell lines in a concentration-dependent manner, which was consistent with the results obtained with CCK-8 (Figure 4B, D). Because of its targeted aPD-L1 peptide, PEG-MP9-aPDL1 was more effective at inducing cell necrosis than PEG-MP9.

Due to its biocompatibility, PEG may serve as a suitable vehicle for the systemic delivery and intracellular release of MP9. Thus, none of the conjugates showed hemolytic activity in RBCs (Figure 4E). Meanwhile, it was found that PEG-MP9-aPDL1 and PEG-MP9 rapidly killed tumor cells, observed in real-time by calcein-AM and PI dual staining (Figure 4F). These results demonstrated that PEG-MP9-aPDL1 had increased oncolytic activity than PEG-MP9, most likely caused by the addition of the aPD-L1 peptide that improved tumor-specific targeting of the oncolytic stapled peptide MP9.

Cancer immunotherapy, which suppresses cancer progression by activating anti-cancer immunity in patients, shows efficacy in treating multiple types of cancers. ICD plays an important role in nanoparticle-mediated cancer immunotherapy 28. The oncolytic tumor cells release immunostimulatory signals commonly known as TAAs and damage-associated molecular patterns (DAMPs), such as ATP, high-mobility group box 1 (HMGB1) and calreticulin (CRT), which may serve as antigenic material for initiating the immune response by antigen presenting cells (APCs). The APCs can migrate to tumor-draining lymph nodes and ultimately initiate the tumor-targeting cytotoxic T lymphocyte (CTL)-dependent antitumor immune response, locally or systemically 29.

As one of the immunogenic onco-cytotoxic agents, oncolytic peptides also exert antitumor activity* in vivo* by promoting immune cell infiltration into tumors 30, 31. At least in part, various oncolytic peptides cause necrosis and then the release of DAMPs, a form of ICD 32. Therefore, we investigated if the stapled peptide MP9 and its conjugates can trigger antitumor immunity by ICD. Treatment with free MP9, PEG-MP9, and PEG-MP9-aPDL1 each triggered ATP release into cell culture media samples in a time- and concentration-dependent manner (Figure 4G-H). Western blot analysis revealed that the levels of cellular HMGB1 protein were reduced after exposure to free peptide MP9 and its conjugates PEG-MP9 and PEG-MP9-aPDL1, respectively (Figure 4I). Immunofluorescence studies revealed that HMGB1 proteins aggregated in the nucleus under normal conditions. After treatment with MP9, PEG-MP9, or PEG-MP9-PDL1, the HMGB1 protein-associated green fluorescence dissipated (Figure 4J). Meanwhile, the confocal laser scanning microscopy (CLSM) images of CT26 and HCT116 stained with CRT antibody indicated that the levels of CRT increased in MP9, PEG-MP9, and PEG-MP9-aPDL1 treatment groups (Figure 4K). Although the free oncolytic stapled peptide MP9 displayed the highest cytotoxicity against tumor cells, the peptide-polymer conjugate that contains the targeting peptide aPD-L1 could cause more robust ICD. Such formulation may afford significant advantages over parent peptide in clinical settings, such as enhanced therapeutic efficacy, reduced side effect(s), and better pharmacokinetic property.

### Biodistribution of PEG-MP9-aPDL1

To assess biodistribution of different polymer chimeras, PEG-MP9-aPDL1, PEG-MP9 and MP9 were labeled with indocyanine green (ICG). When the tumor volume reached about 200 mm^3^, CT26 bearing BALB/c mice were injected in the tail vein with 100 μL (0.5 mg/kg ICG) of ICG-labeled PEG-MP9-aPDL1, ICG-labeled PEG-MP9 or ICG-labeled MP9.

**Figure 5:**
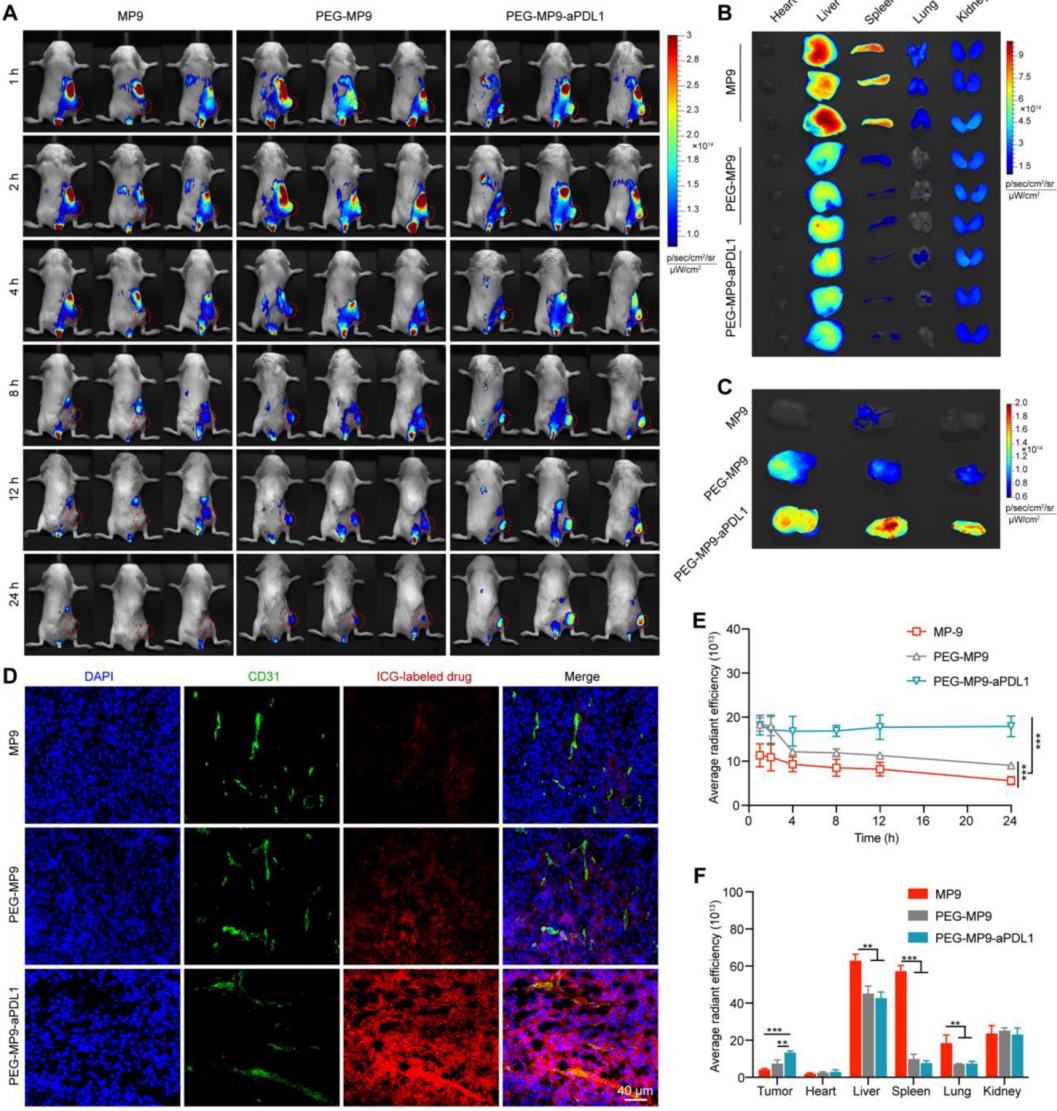
***In vivo* tumor targeting of PEG-MP9-aPDL1. (A)** Female BALB/c bearing CT26 tumor (~200 mm^3^) were given a single intravenous injection of ICG-labeled MP9, ICG-labeled PEG-MP9 or ICG-labeled PEG-MP9-aPDL1 at the ICG dose of 0.5 mg/kg. At 1, 2, 4, 8, 12, and 24 h after injection, mice with *in vivo* ICG fluorescence were imaged by a Bio Imaging Technologies (VISQUE In Vivo Elite). The tumors were shown in red. At 24 h after injection, the mice were sacrificed, and major organs **(B)** and tumors **(C)** were harvested for *ex vivo* imaging. **(D)** Distribution of ICG-labeled MP9, ICG-labeled PEG-MP9 or ICG-labeled PEG-MP9-aPDL1 in cryosectioned tumor tissue samples.** (E)** After injection of ICG-labeled MP9, ICG-labeled PEG-MP9 and ICG-labeled PEG-MP9-aPDL1, the average radiant efficiency of tumors* in vivo* at 1, 2, 4, 8, 12, and 24 h. **(F)** The average radiant efficiency of major organs and tumors. Statistical significance: *p < 0.05, **p < 0.01, and ***p < 0.001 (n = 3).

At 1, 2, 4, 8, 12, and 24 h after injection, *in vivo* near-infrared fluorescence imaging was used to detect ICG distribution *in vivo* (Figure 5A). The fluorescent signal of ICG-labeled PEG-MP9-aPDL1 was distinct observed in the tumor area after 1 h, and it was clearly shown that there was still strong signal at the time of 24 h, which suggested that PEG-MP9-aPDL1 could be retained in the tumor area for a longer time than free MP9 and PEG-MP9 (Figure 5A, E). After 24 h of injection, major tissues and tumors of mice were collected and studied (Figure 5B-C, F). The results showed that PEG-MP9-aPDL1 achieved a significantly higher retention in tumors than other groups. In contrast, the significantly lower fluorescence intensities in the liver, spleen, and lung were found in PEGylated conjugates. The intratumoral distribution of the conjugates was further evaluated by fluorescence imaging of the excised tumor sections. MP9 and PEG-MP9 displayed weak fluorescence signal and distributed at the periphery of the blood vessels (Figure 5D), while PEG-MP9-aPDL1 showed brilliant fluorescence signal and was distributed throughout the tumor section, suggesting PEG-MP9-aPDL1 have a higher ability to penetrate tumors owing to its positive surface charge (Figure 3G) and the targeting ability of aPD-L1 peptide. These data confirmed that the PEG-MP9-aPDL1 could be enriched in the tumor site and exhibited longer retention than free MP9 and PEG-MP9 after systemic injection, providing a great reference for applications of oncolytic peptides *in vivo*.

### PEG-MP9-aPDL1 inhibits tumor growth *in vivo*

**Figure 6:**
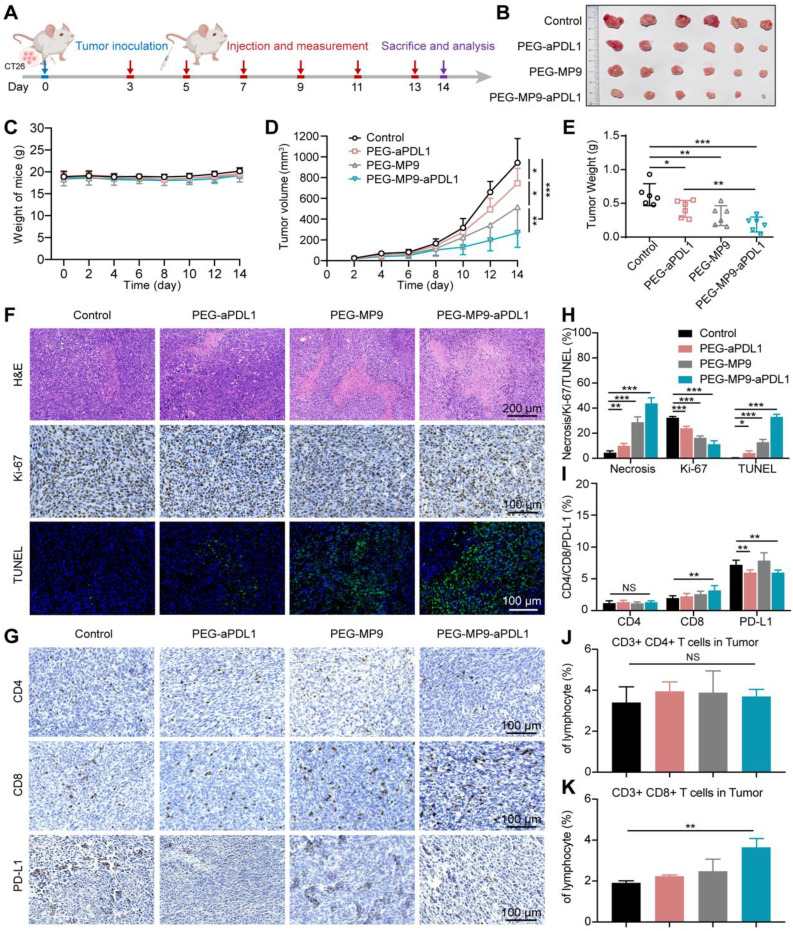
***In vivo***** antitumor efficacy and the evaluation of the immune response in CT26 tumor model. (A)** Schematic illustration of the timeline for the *in vivo* study. **(B)** Images of excised CT26 tumors. **(C)** Body weight of mice bearing CT26 tumors throughout the study. **(D)** Tumor growth curves (n = 6). **(E)** Weight of CT26 tumors. Representative images of H&E, Ki-67, and TUNEL staining of tumor samples collected from different groups **(F)** and image-based quantitative results **(H)** (n = 6). Representative images of tumor samples following immunohistochemical staining for CD4+, CD8+, and PD-L1 **(G)** and quantified results of the images **(I)** (n = 6). Flow cytometry analysis of CD4+ **(J)**, CD8+ T cells **(K)** isolated from CT26 tumors after PBS, PEG-aPDL1, PEG-MP9, and PEG-MP9-aPDL1 treatment (n = 3). Statistical significance: *p < 0.05, **p < 0.01, and ***p < 0.001.

Given that MP9 and its conjugates induced ICD by releasing DAMPs like HMGB1, ATP and CRT, the potential of the PEG-MP9-aPDL1 peptide-polymer conjugate to induce a tumor immunotherapeutic response *in vivo* was investigated following systemic administration in CT26 tumor bearing female BALB/c mice. Approximately three days after inoculation (when the CT26 tumor volumes reached ~30 mm^3^), the tumor-bearing BALB/c mice were divided into groups and administered either vehicle (PBS), PEG-aPDL1, PEG-MP9, or PEG-MP9-aPDL1 via intravenous injection once every two days (adjusted to an equivalent dose of 3 mg/kg MP9 peptide) (Figure 6A). During the treatment, there was no apparent body weight loss or abnormal behavior in any of the groups (Figure 6C), indicating that potential side effects were minimal. Serum samples showed no significant hemolytic side effect in PEG-MP9-aPDL1 or PEG-MP9 groups when compared with the control group, indicating that polymeric materials reduced potential hematological toxicity hemolytic toxicity (Figure S16).

The PEG-MP9-aPDL1 treatment was the most effective at suppressing tumor growth. However, the tumors were not completely eradicated in the PEG-MP9-aPDL1 group at the final endpoint of the study (Figure 6B). Although PEG-aPDL1 and PEG-MP9 possessed certain therapeutic effects, initial tumor growth was only slightly inhibited by these peptides (Figure 6D-E). Pathological and immunohistochemical examination of tumor samples indicated that PEG-MP9-aPDL1 and PEG-MP9 treatment led to significantly increased necrotic lesions, TUNEL-positive cells, and decreased Ki-67 positive cells, when compared to the vehicle control (Figure 6F, H). A lower level of tumor cell necrosis and apoptosis was observed with PEG-aPDL1 treatment. Collectively, PEG-aPDL1 did not significantly affect tumor growth *in vivo*. No obvious major organ damage was observed in the PEG-MP9-aPDL1 group, same with the other treatment groups, indicating that the combination therapy potentially had a good margin of safety (Figure S17).

To identify the factor(s)/contributor(s) for improved efficacy, the ICD of tumors was studied by immunofluorescent staining. We observed the dramatic increase of HMGB1 excretion and CRT exposure in tumor tissues treated with PEG-MP9-aPDL1 than other groups (Figure S18). Those DAMPs were important factors in boosting antitumor immunity. We further investigated the intratumoral level of DCs (CD11c+ cells). The immunofluorescent staining displayed the increased expression of CD11c, which suggested the accelerated infiltration of DCs after PEG-MP9-aPDL1 mediated ICD (Figure S19). On the other hand, CD4+/CD8+ T cell recruitment and tumor infiltration were investigated (Figure S20). In the PEG-MP9-aPDL1 group, the anti-CD8 antibody staining identified the presence of a comparably large number of T cells (stained brown) in the tumor, suggesting their potential contribution of cellular immunity (Figure 6G, I). In flow cytometry studies, the PEG-MP9-aPDL1 group had increased CD3+CD8+ T cell population in tumors when compared to the PEG-aPDL1 and PEG-MP9 groups, while no change was observed with the CD3+CD4+ T cell population (Figure 6J-K). The content of CD4+ and CD8+ T cells increased lightly in the spleen after PEG-MP9-aPDL1 treatment (Figure S21)**.** The effects of PEG-MP9-aPDL1 on tumor growth and CD8+ T cell infiltration suggested that this peptide-polymer conjugate may also promote antitumor immunity. To test this hypothesis in CRC models, mice carrying CT26 tumors treated with PEG-MP9-aPDL1 also received anti-CD8 antibodies to deplete the CD8+ T cells (Figure S22A). As expected, CD8+ T cell depletion in PEG-MP9-aPDL1 treated mice did not inhibit initial CT26 tumor progression (Figure S22B-E). We also found that after anti-CD8 antibody treatment, the necrotic area of tumor was reduced, the degree of apoptosis was decreased, and the infiltration of CD4+ and CD8+ T cells was also reduced, observed using immunohistochemical methods (Figure S22F-G).

Because PEG-MP9-aPDL1 incurred ICD and DAMPs, the recruited CD8+ T cells could be blocked by immune checkpoint inhibitor anti-PD-L1 peptide that contributes to the tumor immunosuppressive microenvironment. This validated the necessity of combining MP9 and anti-PD-L1 peptides and indicated that PEG-MP9-aPDL1 could be a promising antitumor agent in the CT26 model.

## Discussion

The high degree of intratumoral heterogeneity (ITH) of CRC is one of the major factors contributing to immunotherapeutic responses in patients 33. Oncolytic peptides can directly kill tumor cells that producing drug resistance and drive ICD, exerting a powerful anti-tumor immune response independently of ITH 34. Combining oncolytic peptides with immune checkpoint blockade (ICB) for CRC may enhance the effect of oncolytic immunotherapy, thereby expanding the usage of immunotherapy in patients with CRC 35. Meanwhile, PD-L1 antibody is able to effectively block the interaction of PD-1/PD-L1 to overcome immune escape. However, PD-L1 antibodies have some limits associated with the production cost, immunogenicity, and low tissue penetration. Recently, PD-1/PD-L1 antagonists based on small molecular peptides have received much attention due to the advantages of lower manufacturing cost, modifiable structure, tumor penetration, and reduced immunogenicity 36, 37. With the boom of new peptide-based therapies, the ability to modify and deliver peptides have recently gained great significance in both medicinal chemistry optimization and drug development 38-40.

Peptide-polymer conjugates have good biocompatibility, and are becoming a favorable pattern to develop new peptide therapies and diagnostic reagents. Through intravenous administration, peptide-polymer conjugates can not only prolong drug serum half-lives and increase drug tissue permeability 41, but also enhance drug accumulation within the tumors through the combined effect of enhanced permeability and retention (also known as EPR) to achieve an effective systemic effect 42, 43. Notably, it is also popular that use a carrier molecule to conjugate with cytotoxic drugs or peptides. The polymer-drug system, including carboxymethylcellulose, doxorubicin, and functionalized with peptide RGD and L-arginine, realized the targeting chemotherapy for cancers 44. Using a multivalent polymer conjugated with an oncolytic peptide also could enhance the membrane lytic property and impose ICD in 4T1 tumor-bearing mice 30. In addition, the CpG adjuvant and oncolytic peptides LTX-315 co-loaded core-shell nanoparticles exhibited strong immune response, but the synthesis steps were relatively cumbersome and needed the additional anti-PD-L1 antibody to achieve long-term immune memory protection 45.

In this research, stapled MP derivatives have been successfully produced via the standard SPPS strategy and ring-closing metathesis (RCM) reaction. *In vitro* results suggest that the derivative MP9 displays improved helicity levels, greater protease stability, and enhanced antitumor activity when compared with the original MP peptide, making it a promising candidate for developing novel cancer therapeutic. To overcome increased hemolytic activity and other obstacles associated with systemic administration, MP9 was transformed into a bifunctional peptide (MP9-MMP-2-aPD-L1)-polymer conjugate using high polymer material PEG. Initially, we used both polymer material 4-arm PEG and 8-arm PEG to synthesize peptide-polymer conjugates. Although the antiproliferative activity of 8-arm PEG increased compared to 4-arm PEG (Figure S23), the hemolytic activity was also observed (Figure S24). As a result, the 4-arm PEG was selected as the delivery backbone. Since MMP-2 was enriched in TME (Figure S25), MMP-2 linker could be cleaved within the tumor microenvironment, and the free D-type anti-PD-L1 peptide can bind to the PD-L1 receptor on tumor cells to block the potential impact of tumor cell immune escape. Further, the exposed stapled MP9 peptide quickly killed tumors, thus enhancing the oncolytic immunotherapeutic response. In contrast to previous systemic delivery vehicle based on oncolytic peptide, PEG-MP9-aPDL1 represents a versatile treatment platform for CRC by integrating different peptides with multiple therapeutic mechanisms into one polymer chimera. We anticipate that this peptide-polymer conjugate would be promising in more and more biomedical research and clinical applications in the near future.

## Conclusion

In summary, we present an all-in-one polymer chimera based on stapled oncolytic peptide for targeted oncolysis and synergistic ICB therapy, addressing the insensitivity of CRC to anti-PD-L1 immunotherapy due to neoantigen deficiency. By stapling chemical methodology, we obtained a wasp venom-derived peptide MP9 with pronounced oncolytic effect and enhanced proteolytic stability. To realize the systemic administration and improved therapeutic activity of MP9 *in vivo*, we developed the polymer chimera PEG-MP9-aPDL1 that exhibited favorable biosafety, durable circulation stability, effective tumor targeting, and synergistic oncolytic immunotherapeutic efficacy in a mouse model for CRC. Our findings suggest that this polymer chimera offers a promising paradigm for enhancing efficacy of immune checkpoint inhibitors and is valuable to advance oncolytic peptide-related basic research towards future clinical application.

## Methods

### Materials and reagents

All reagents and solvents were purchased from Adamas-beta, GL Biotech, Energy Chemical or Sinopharm Chemical Reagent Co. Ltd. Rink Amide resin (loading 0.34 mmol/g) was purchased from Tianjin Nankai Hecheng Science & Technology Co. Ltd. 4-arm PEG-maleimide (MW 5K Da) and 8-arm PEG-maleimide (MW 10K Da) was supplied from Jinpan biotech (Shanghai, China).

### Cell lines

HUVECs were purchased from Lifeline Cell Technology and cultured in completed endothelial cell medium (Lifeline^®^ Cell Technology, Frederick, MD). CRC cell lines HCT116 and CT26 were purchased from Cell Bank of Shanghai institute of Cell Biology, Chinese Academy of Sciences, and cultured in McCoy's 5A (Gibco, 16600082) and 1640 (Gibco, 2192717) medium, respectively, with 10% fetal bovine serum (FBS, Gibco, 10091148) and 1% penicillin-streptomycin (HyClone, SV30010). All cell lines used in the study were maintained in a humidified incubator (Thermo Fisher, USA) containing 5% CO_2_ at 37 ℃.

### Analytical methods

^1^H NMR spectra was obtained on a Bruker Avance 500 MHz NMR Spectrometer (Germany). The chemical shifts of protons are given on the *δ* scale, ppm, with tetramethyl silane as the internal standard. All NMR experiments were conducted at room temperature unless otherwise stated.

Analytical HPLC was run on an Agilent 1260 Infinity LC instrument using an analytical column (ZORBAX Eclipse XDB 80 Å C18, 4.6 ×150 mm, 3.5 μm particle size, flow rate 1.0 mL/min, r.t.). Analytical injections were monitored at 214 nm. All separations used a mobile phase of 0.1% trifluoroacetic acid (TFA) (v/v) in water (solvent A) and 0.1% TFA (v/v) in CH_3_CN (solvent B), with a linear gradient of 5-65% solvent B in 30 min at room temperature. Semipreparative HPLC was run on a Waters Auto Purification System (USA) instrument using a semipreparative column (XBridge Prep C18, 19 × 250 mm, 10 μm particle size, flow rate 20 mL/min). Solution A was 0.1% TFA in water, and solution B was 0.1% TFA in CH_3_CN. Gradient was a linear gradient of 20-20% B over 5 min and then a linear gradient of 20-60% B over 50 min. The linear and stapled peptides were dissolved in the H_2_O to a final concentration of 50 μM, respectively.

### Synthesis of peptide MP and its derivatives

All peptides were synthesized by standard Fmoc solid-phase peptide synthesis (SPPS) using Fmoc chemistry on the Rink Amide resin (initial loading = 0.34 mmol/g). Briefly, 735 mg resin was swelled with dichloromethane (DCM) for 20 min, followed by washing with N, N-dimethylformamide (DMF) 3 times at 35 ℃. Fmoc deprotection of resin was using 20% piperidine/DMF/0.1 M Oxyma pure twice (5 min × 2). For each step of amino acid coupling, the resin was coupled with amino acid (1 mmol), N, N'-diisopropylcarbodiimide (DIC, 1 mmol) and Oxyma pure (1 mmol) in N-Methyl-2-pyrrolidone (NMP, 6 mL) solvent. The reaction was conducted in a constant temperature shaker for 20 min at 60 ℃. Then, the resin was washed with DMF (3 times), DCM (3 times), and DMF (3 times), respectively. Notably, the peptide couplings of Fmoc-(S)-2-(4-pentenyl) Ala-OH (Fmoc-S_5_-OH, 0.5 mmol) were carried out over a single 2 h coupling cycle at 60 ℃. The deprotection, washing, coupling, and washing steps were assembled until all the amino acid residues were assembled.

After the resin was washed it was treated with 6 mL solution of acetic anhydride and pyridine (1:1) for 20 mins. Ring-closing metathesis (RCM) reaction was realized using first generation Grubbs' catalyst (10 mM) in dry dichloroethane (DCE) solution for 2 h at 37 ℃ (2 times). After the peptide-resin was washed with DMF (5 times) and DCM (5 times), a mixture of 95% TFA, 2.5% triisopropylsilane (TIPS) and 2.5% water (volume ratio) was added to cleave peptides from resins for 2 h at room temperature. Then, TFA was evaporated by blowing with N_2_. The crude peptides were obtained by precipitation with 40 mL of cold diethyl ether and centrifugation at 4000 rpm for 3 min (3 times). Finally, the crude products were allowed to air dry and purified by reverse phase high-performance liquid chromatography (RP-HPLC) to give the final products. All peptides were confirmed by mass spectrum (MS) and possess a purity of at least 95% (Figure S26-63 and Table S1).

### Synthesis and characterization of peptide-polymer conjugates

Peptides with additional Cys residue at the N-terminal and 4-arm PEG-maleimide were dissolved in PBS (pH 7.4) at a molar ratio of 8:1, respectively. The reaction mixture was stirred at room temperature overnight. Then the products were isolated by ultrafiltration tubes (10K Da NMWL, Millipore) at 4000 g for 15 min (five times), and the pure powders were acquired after freeze-drying.

The products were confirmed by ^1^H nuclear magnetic resonance (^1^H NMR) spectra. The particle size and zeta potential of prepared peptide-polymer conjugates PEG-MP9-aPDL1, PEG-MP9 and PEG-aPDL1 were measured by Zetasizer Nano ZS instrument (Malvern, UK). And the morphology of conjugates was characterized by TEM.

The concentration of peptides was determined by Ultra-micro spectrophotometer at 206 nm. The conjugation efficiency was calculated according to the following formula based on a calibration curve obtained by measuring the absorption of a series of peptides solutions with known concentrations (0.01-2 mg/mL):

Conjugation efficiency (wt%) = 
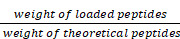
 × 100%

### Fluorescent labeling of peptide-polymer conjugates

The ICG-DBCO purchased by Xi'an Ruixi Biological Technology Co. Ltd was dissolved in the DMSO and the standard curve of ICG-DBCO at the absorption peak of 821 nm was obtained by ultra-micro-ultra-ultraviolet spectrophotometer. Peptides Cys-MP9-MMP-2-aPD-L1 and Cys-MP9 with additional Lys (N_3_)-residue at the C-terminal reacted with 4-arm PEG-maleimide conducting as above protocol to get peptide-polymer conjugates PEG-K (N_3_)-MP9 and PEG-K (N_3_)-MP9-aPDL1. The ICG-DBCO was reacted with the peptide-polymer conjugates at a molar ratio of 8:1 by click-chemical reaction for 12 h at 30 ℃. Then the products were isolated by ultrafiltration at 4000 g for 15 min (five times). The pure powders were obtained after freeze-drying.

### Cell viability assay

Cell viability was examined using the Cell Counting Kit-8 (CCK-8, Meilunbio, MA0218). CT26 cells and HCT116 cells were seeded into 96-well plates at a density of 5 × 10^3^ cells/well and incubated overnight. The peptides and conjugates with various concentrations were added for 24 h. Then the media was replaced with fresh serum-free media containing 10% CCK-8 solution and incubated for another 2 h at 37 ℃. The absorbance was detected by Cytation 5 (BioTek, USA) microplate reader under 450 nm as previously described 13 and the half maximal inhibitory concentration (IC_50_) was calculated by GraphPad Prism 8.

### CD spectroscopy study

The circular dichroism (CD) spectra were used to explore the secondary structure of peptides on BRIGHT TIME Chirasca (Applied photophysics, Britain) spectropolarimeter. Peptides and conjugates in aqueous solution were recorded at 25 ℃ in a quartz cell of 1 mm and 0.5 mm path length, respectively. All spectra were converted to a uniform scale of molar ellipticity after background subtraction. The curves were smoothed using standard parameters. In addition, according to the ellipticity of the peptide's spectrum at 222 nm and the number of amino acids in the peptide sequence, the helicity of each peptide was calculated using the literature equation 46. Percent helicity was calculated by the follow equation:

α = ([θ]_222_) / ([θ]_max_) × 100%

[θ]_222_ is the molar ellipticity of 222 nm; [θ]_max_ = (-44,000 + 250T) × (1 - k/n), k = 4, where n is the numbers of amino acids and T = 25 ℃.

### Hemolysis assay

The hemolytic activities of the peptides and peptide-polymer conjugates were evaluated by determining hemoglobin release from suspensions of mice blood. Fresh erythrocytes were obtained from the whole blood by centrifuging at 4 ℃ 1000 g for 30 min and washing with PBS 2 times. Then erythrocytes were diluted with PBS at the volume ration of 1:10. The 96-well plate was added to 100 μL erythrocyte suspension per well and incubated with peptides and conjugates solution with different concentrations for 1 h at 37 ℃, following centrifuged at 1000 rpm for 10 min. The supernatants were obtained and monitored by measuring absorbance at 570 nm (BioTek Cytation 5, USA) as previously described 13. 0.1% (v/v) Triton X-100 was used as a positive control and PBS was used as a negative control.

### LDH release assay

Using cytotoxicity lactate dehydrogenase release assay Kit-WST (DOJINDO, CK-12) to perform LDH release. CT26 cells and HCT116 cells were plated in triplicate wells into 96-well plates at a density of 5 × 10^3^ cells/well, respectively. After culturing overnight, the medium in the wells was replaced with fresh medium containing peptides or conjugates at different concentrations and cells without drug were treated as control. Lysis buffer was added as a positive control for 30 min at room temperature after 24 h incubation. Finally added the stop solution and the absorbance was measured at 490 nm by a microplate reader (BioTek Cytation 5, USA).

### LIVE/DEAD cell viability/cytotoxicity

CT26 cells and HCT116 cells were plated in triplicate wells into 96-well plates at a density of 1 × 10^4^ cells/well. After culturing overnight, cells were washed twice with PBS and were labeled with 2 μM calcein-AM (Ex/Em = 488/515 nm) for 15 min. Then the cells were treated with peptides of different doses containing 8 μM PI (Ex/Em = 535/617 nm). The cells were photographed for varying time points by Operetta CLS High Content Analysis System (PE, USA).

### Proteolytic stability experiment

The peptides were dissolved in H_2_O to a final concentration at 2 mM, respectively. α-Chymotrypsin was dissolved in PBS (0.5 ng/μL, containing 2 mM CaCl_2_, pH 7.4). Then, the peptide solutions (50 μL) were incubated with α-chymotrypsin solution (950 μL) at 37 ℃. 100 μL of digestion mixture was taken at the different time and then quenched with 20 μL of hydrochloric acid (1 M). The percent residual peptide was monitored by HPLC at 214 nm. Each experiment was performed in triplicate.

### Real-time and label-free cell growth inhibitory

The xCELLigence RTCA systems (Agilent, USA), based on electrical impedance, allows real-time and label-free measurement of cell proliferation and cytotoxicity. Moreover, the obtained cell index reflects a comprehensive characterization including cell adhesion and spreading. The background signal was detected with cell culture medium/well. For proliferation assay, 5 × 10^3^ CT26 cells per well were seeded on E-Plate 16 and incubated overnight. The cells were treated with or without MP9 with various concentrations for 12 h. Then cell index values were calculated using the RTCA software.

### *In vitro* binding assays by microscale thermophoresis (MST)

The binding affinity of aPD-L1 peptide to PD-L1 protein (Novoprotein, China) was determined by microscale thermophoresis (MST). PD-L1 protein was labeled using “Monolith NT.115^TM^ protein Labeling Kit RED”, which reacts efficiently with the primary amines of protein to form highly stable dye-protein conjugates via N-hydroxysuccinimide (NHS)-ester chemistry. In the MST experiments, the concentration of PD-L1 was keep constant, while the aPD-L1 peptide was serially diluted. And the PD-L1 was mixed with 16 titration series of aPD-L1 peptide at the volume ration of 1:1. After a short incubation, the mixture was loaded into MST NT.115 standard glass capillaries and measured by Monolith NT.115 (NanoTemper, Germany).

### Cleavage of peptide MMP-2-aPD-L1 by an exogenous MMP-2 trigger

To analyze the enzymatic cleavage of matrix metalloproteinase (MMP-2)-sensitive peptide (MP9-MMP-2-aPD-L1), MMP-2-mediated cleavage was studied in the presence of MMP-2 (R&D systems, USA) in PBS (pH 7.4). MMP-2 was activated with the 2.5 mM 4-aminophenylmercuric acetate (APMA) solution at 37 ℃ for 1 h. After incubation for 5, 10, 15, 20, 30, and 60 min, the aliquots were quenched with hydrochloric acid and analyzed by HPLC and MS.

### Cell uptake

Cells were seeded (5 × 10^3^ cells/well) in 96-well plates and treated with FITC-labeled conjugates PEG-MP9 and PEG-MP9-aPDL1 at IC_50_ concentrations for 10, 30, and 60 min. Then, the cells were washed with cold PBS 3 times and fixed in 4% paraformaldehyde (Beyotime, P0099) for 20 min, and stained with Hoechst 33342 to label the cell nuclei. The fluorescence intensity of cells was measured by Operetta CLS High Content Analysis System (PE, USA).

Cells were seeded in a glass-bottom dish at a density of 5 × 10^4^ cells/well and incubated overnight. The cells were treated with FITC-labeled conjugates PEG-MP9 and PEG-MP9-aPDL1 at IC_50_ concentrations for 30 min. Then, the cells were washed with cold PBS 3 times and fixed in 4% paraformaldehyde (Beyotime, P0099) for 20 min, and stained with Hoechst 33342 to label the cell nuclei. The cell uptake of conjugates was observed by GE DeltaVision OMX SR (GE, USA).

### Observation of MP9 and PEG-MP9-aPDL1 effect using NanoLive 3D cell explorer

Cells were seeded in a glass-bottom dish at a density of 5 × 10^4^ cells/well and incubated overnight. Cells were washed twice with PBS and then treated with MP9 and PEG-MP9-aPDL1 dissolved in PBS, respectively. Using brightfield of the NanoLive 3D Cell Explorer to investigate the dynamic changes of cells after treated with MP9 and PEG-MP9-aPDL1.

### ATP release assay

Cells were seeded (5 × 10^3^ cells/well) in 96-well plates and treated with peptide MP9 and its conjugates at different concentrations for 1 h. All experiments were performed in duplicate at a total of 3 times. Negative controls were untreated cells exposed to a serum-free medium alone. The supernatant of the treated cells was detected using an enhanced ATP assay kit (Beyotime, S0027). Samples were analyzed using a luminometer (Tecan Spark, Switzerland) according to the manufacturer's protocol.

### Immunofluorescence staining of CRT

CT26 cells and HCT116 cells were seeded in a glass-bottom dish at a density of 5 × 10^4^ cells/well and incubated overnight. The cells were treated with MP9, PEG-MP9, and PEG-MP9-aPDL1 for 6 h (MP9 concentration: 10 μM in CT26; 20 μM in HCT116). Cells incubated with only medium served as a control. Then, the cells were washed with cold PBS 3 times and fixed in 4% paraformaldehyde (Beyotime, P0099) for 20 min. Nonspecific binding sites were blocked by 1% BSA at room temperature for 1 h, followed by incubation with anti-Calreticulin-Alexa Fluor 647 antibody (Abcam, ab196159) for 1 h, and stained with Hoechst 33342 to label the cell nuclei. Surface detection of CRT was observed by GE DeltaVision OMX SR.

### Immunofluorescence staining of HMGB1

CT26 and HCT116 cells were seeded in glass-bottom dish at a density of 5 × 10^4^ cells/well and incubated overnight. The cells were treated with MP9, PEG-MP9, and PEG-MP9-aPDL1 for 6 h (MP9 concentration: 10 μM in CT26; 20 μM in HCT116). Cells incubated with only medium served as a control. Then, the cells were washed with cold PBS 3 times and fixed in 4% paraformaldehyde for 20 min and then permeabilized with 1% Triton X-100 for another 5 min. Nonspecific binding sites were blocked by 1% BSA at room temperature for 1 h, followed by incubation with anti-HMGB1 antibody (Abcam, ab227168) at 4 ℃ for 16 h, and then incubated with an Alexa Fluor 488-conjugated secondary antibody (Abcam, ab150077) for 2 h at room temperature after washing three times with PBS. Finally, the cells were stained with Hoechst 33342 and detected by GE DeltaVision OMX SR.

### Western blot assay

CT26 cells and HCT116 cells were treated with MP9, PEG-MP9, and PEG-MP9-aPDL1 for 6 h, respectively. Cells were collected and lysed in ice-cold NP-40 buffer containing protease inhibitors and then were centrifuged at 4 ℃ and 15000 rpm for 10 min. The supernatant was collected as samples. The total protein was quantified with BCA assay kit (Beyotime, P0010S). All samples were separated in 12.5% SDS-PAGE and transferred onto polyvinylidene difluoride (PVDF) membranes (GE, 10600021). The PVDF membranes were blocked in Tris-buffered saline containing 1% Tween-20 (TBST, pH 7.4) with 5% non-fat milk for 1 h. Following, the members were incubated with primary antibodies at 4 ℃ overnight. After washing with TBST 3 times, the membranes were incubated with secondary antibodies for 1 h. Detection was performed by gel Imaging System (Tanon, China) and the bands were analyzed by densitometry using Image J software (NIH, USA). β-actin was used as an internal control.

### Biodistribution

Five-week-old female BALB/c mice were injected with 1 × 10^6^ CT26 cells subcutaneously into the right hind limb of mice. When the tumor volume reached about 200 mm^3^, three mice from each group were injected through the caudal vein with 100 μL (0.5 mg/kg ICG) of ICG-MP9, ICG-PEG-MP9, and ICG-PEG-MP9-aPDL1, respectively. After 1, 2, 4, 8, 12, and 24 h, the mice were anaesthetized and imaged under the Bio Imaging Technologies (VISQUE In Vivo Elite) using near-infrared fluorescence imaging system for ICG fluorescence distribution. Following the* in vivo* imaging, the mice were sacrificed and the tumor, hearts, livers, spleens, lungs and kidneys were harvested for *ex vivo* bioluminescent imaging. The results were analyzed by the software VISQUE Clevue.

### *In vivo* antitumor therapy

Five-week-old female BALB/c mice were purchased from Shanghai Model Organisms Center, Inc and kept under SPF conditions. The animal experiments were approved by Committee on the Ethic of Animal experiment of the Shanghai Model Organisms Center, Inc. 8×10^5^ CT26 cells suspended in 100 μL PBS were subcutaneously inoculated into the right flanks of mice. The tumor volume was monitored periodically by a vernier caliper and calculated using the following formula: volume (mm^3^) = (length × width^2^)/2, where length is the largest tumor diameter and width is the smallest tumor diameter.

When the average volume of tumors reached about 30 mm^3^, the mice were randomly divided into 4 groups (n = 6) and injected with PBS (vehicle control), PEG-aPDL1, PEG-MP9, and PEG-MP9-aPDL1 intravenously. All formulations were dissolved in PBS (3 mg/kg MP9 peptide equivalence) and administered every 2 days for 6 times in total. At the end of the experiment, the animals were sacrificed and tumors were collected for further study. The hearts, livers, spleens, lungs, and kidneys were stained for MMP-2 and hematoxylin and eosin (H&E). The tumors were stained for H&E, Ki-67, TUNEL, CD4, CD8, PD-L1, HMGB1, CRT, CD11c, and MMP-2.

### Analyses of T cell population in tumor

Spleens and tumor tissues were made into single-cell suspensions, and the splenocytes and tumor-infiltrating lymphocytes were quantitatively analyzed by flow cytometry after immunofluorescence staining. In brief, tumor tissues were harvested and digested with Tumor Dissociation Kit (Miltenyi Biotec, 130-096-730). Cells were stained by the addition of a cocktail of fluorescence conjugated anti-Mouse CD3e-PE (biogems, 05122-60), anti-Mouse CD4-FITC (biogems, 06122-50), anti-Mouse CD8a-APC (biogems, 10122-80) antibodies according to the manufacture's protocols for flow cytometric analyses.

### *In vivo* CD8+ T cell depletion

To deplete CD8+ T cells, BALB/c mice received anti-CD8a monoclonal antibody i.p. (BioXcell, BE0117). The antibody (200 μg per mouse) was diluted in PBS. Injections were started one day before the first PEG-MP9-aPDL1 injection and were continued every 3 days for 4 times in total.

### Statistical Analysis

Using GraphPad Prism 8.0.1 statistical analysis software (La Jolla, CA, USA) to analyze data. The data were expressed as the mean ± standard deviation (SD) from at least three-independent experiments. Student's t-test or two-way ANOVA, followed by Tukey's test was applied for comparison between two groups or among multiple groups, respectively: *p < 0.05, **p < 0.01, ***p < 0.001.

## Supplementary Material

Supplementary figures and tables.

## Abbreviations

APCs: antigen presenting cells
APMA: 4-aminophenylmercuric acetate
CD: circular dichroism
CLSM: confocal laser scanning microscope
CRC: colorectal cancer
CRT: calreticulin
CTLs: cytotoxic lymphocytes
DAMPs: damage-associated molecular patterns
DCE: dichloroethane
DCM: dichloromethane
DIC: N, N'-diisopropylcarbodiimide
DLS: dynamic light scattering
DMF: dimethylformamide
dMMR: deficient mismatch repair
EPR: enhanced permeability and retention
H&E: hematoxylin and eosin
HMGB1: high-mobility group box 1
HNMs: hydrophobicity network maps
HUVECs: primary human umbilical vascular endothelial cells
ICB: immune checkpoint blockade
ICD: immunogenic cell death
ICG: indocyanine green
ITH: intratumoral heterogeneity
LDH: Lactate dehydrogenase
LI: lyticity Index
MMP-2: matrix metalloproteinase-2
MP: mastoparan
MSI-H: high microsatellite instable
MST: microscale thermophoresis
MTBE: methyl tert-butyl ether
NHS: N-hydroxysuccinimide
NMP: N-Methyl pyrrolidone
NMR: nuclear magnetic resonance
PD-1: programmed cell death protein-1
PD-L1: programmed cell death 1-ligand 1
PEG: polyethylene glycol
PI: propidium iodide
pMMR: mismatch repair-proficient
PVDF: polyvinylidene difluoride
RBC: red blood cells
RCM: ring-closing metathesis
RP-HPLC: reverse phase high-performance liquid chromatography
RTCA: real time cell analysis
SD: standard deviation
SPPS: solid-phase peptide synthesis
TAAs: tumor-associated antigens
TEM: transmission electron microscopy
TFA: trifluoroacetic acid
TI: therapeutic index
TIPS: triisopropylsilane
TME: tumor microenvironment
