# Supplementary material for: Polymer chimera of stapled oncolytic peptide coupled with anti-PD-L1 peptide boosts immunotherapy of colorectal cancer
Source: Theranostics. 2022 Apr 24;12(7):3456–73. doi: 10.7150/thno.71129 (PMC9065177; doi:10.7150/thno.71129)
Supplement: Supplementary file 1 — Supplementary figures and tables. [file thnov12p3456s1.pdf]

Supporting Information

Polymer chimera of stapled oncolytic peptide coupled with anti-PD-L1 peptide boosts immunotherapy of colorectal cancer

Lu Lu[1*], He Zhang[1,2*], Yudong Zhou[1,3*], Jiayi Lin[1], Weidong Gao[1], Ting Yang[1], Jinmei Jin[1], Lijun Zhang[1], Dale G. Nagle[1,4], Weidong Zhang[1], Ye Wu[1✉], Hongzhuan Chen[1✉], and Xin Luan[1✉]

1. Shanghai Frontiers Science Center for Chinese Medicine Chemical Biology, Institute of Interdisciplinary Integrative Medicine Research, Shanghai University of Traditional Chinese Medicine, Shanghai, 201203, China
2. School of Pharmacy, Fudan University, Shanghai, 201203, China.
3. Department of Chemistry and Biochemistry, College of Liberal Arts, University of Mississippi, University, MS, 38677-1848, USA
4. Department of BioMolecular Sciences and Research Institute of Pharmaceutical Sciences, School of Pharmacy, University of Mississippi, University, MS 38677-1848, USA

*These authors contributed equally to this work.

✉Corresponding author: Ye Wu, wuye@shutcm.edu.cn; Hongzhuan Chen, hongzhuan_chen@hotmail.com; Xin Luan, luanxin@shutcm.edu.cn.

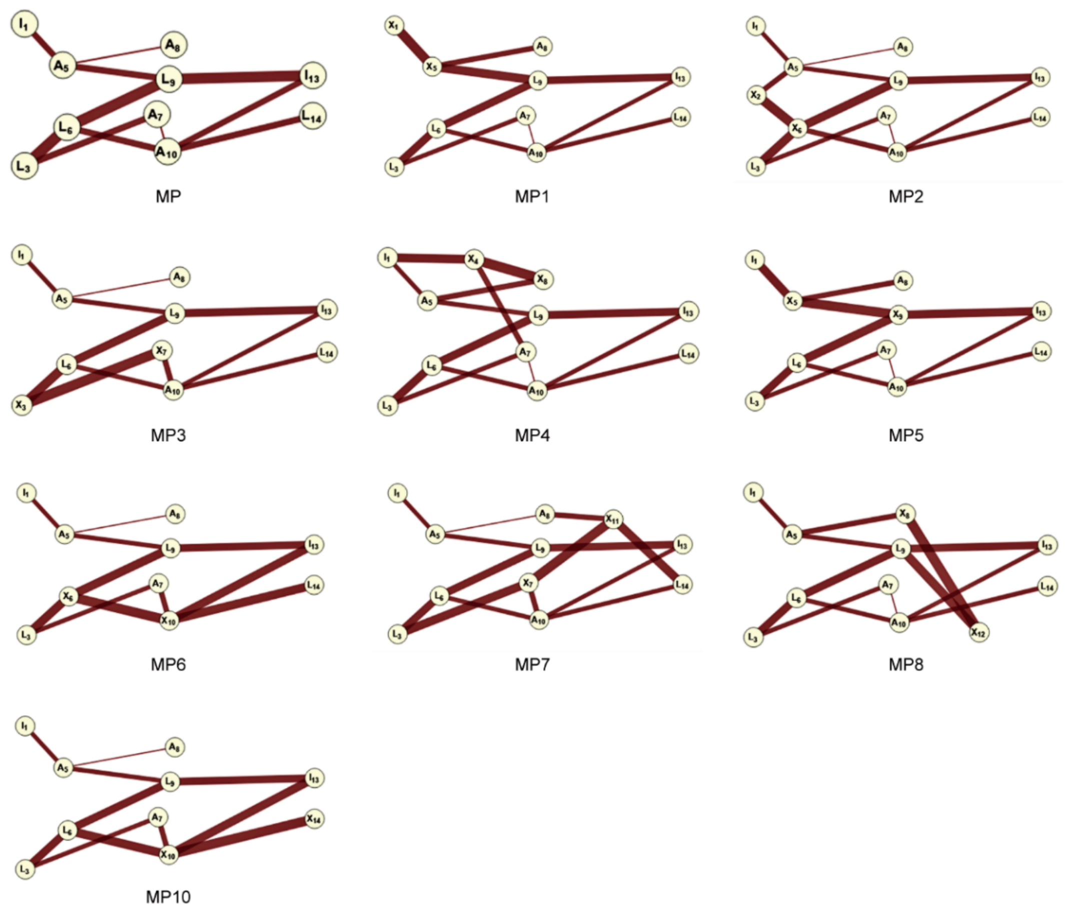

Figure S1. The hydrophobicity network maps (HNMs) of MP, MP1-8, and MP10.

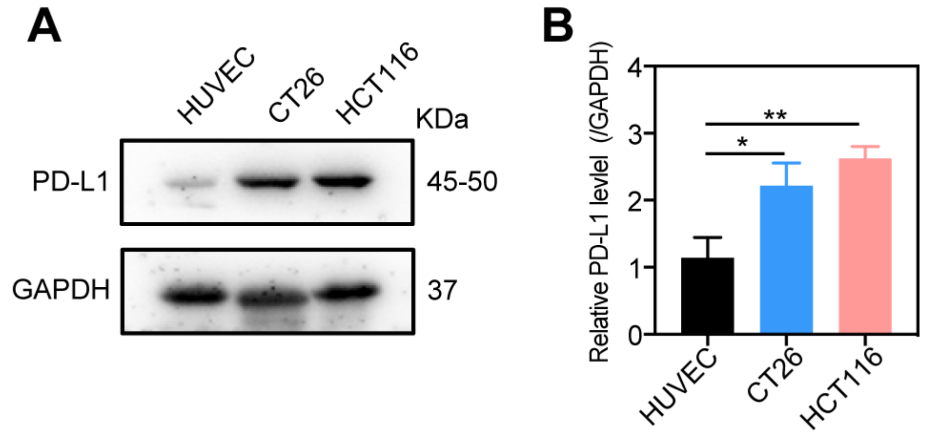

Figure S2. The expression of PD-L1 protein in HUVEC, CT26, and HCT116 cells. Data are presented as mean ± s.d.; n = 3. Statistical significance: *$p < 0.05$, and **$p < 0.01$.

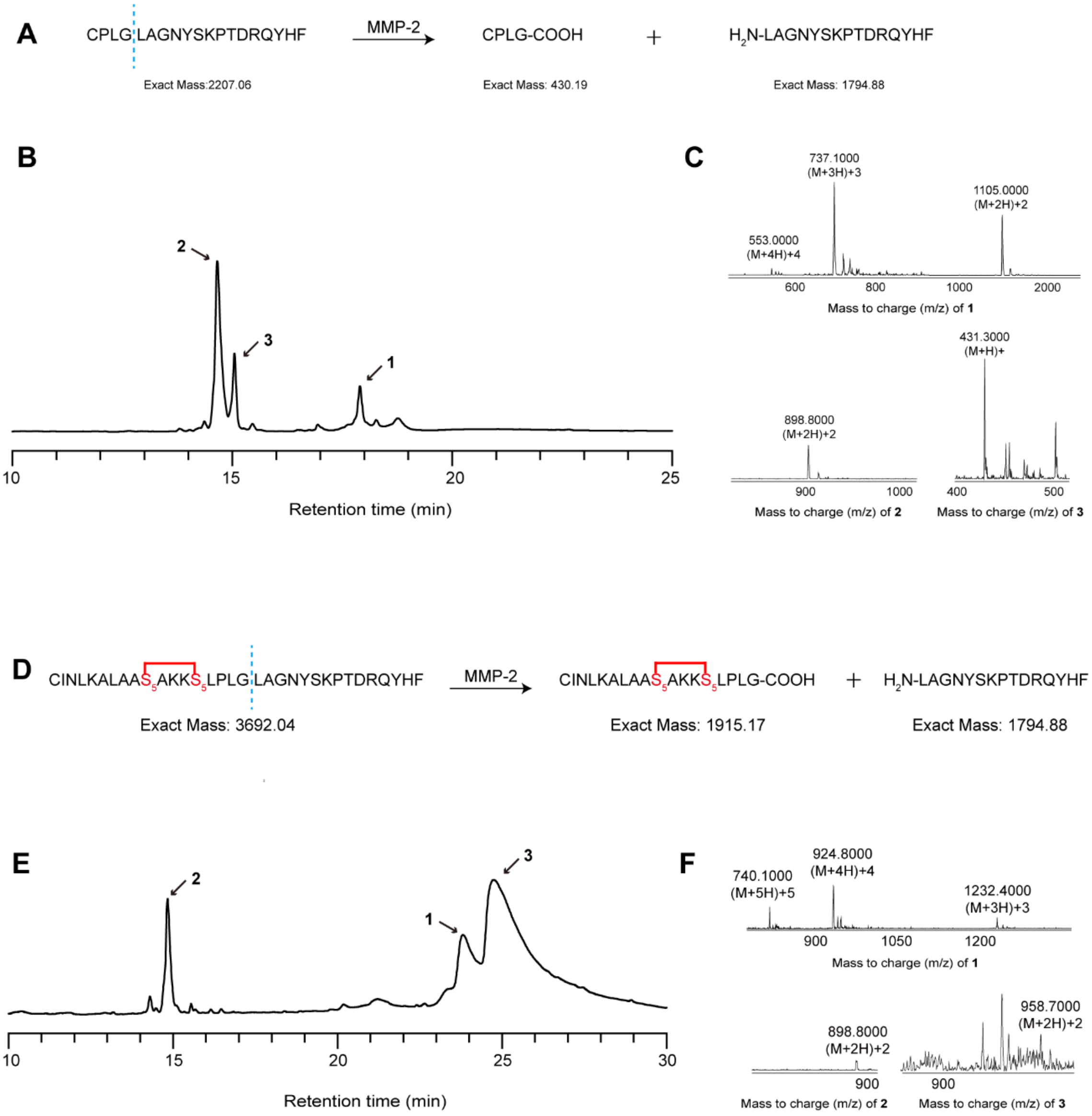

Figure S3. Proteolytic-responsiveness of the peptide Cys-MMP-2-aPD-L1 (**A**) and Cys-MP9-MMP-2-aPD-L1MMP-2 (**D**) in the presence of exogenous MMP-2 (2 μg/mL) for 10 min. Both peptides possess the MMP-2 cleavable linker (-PLGLAG-). HPLC (**B**) and MS (**C**) of Cys-MMP-2-aPD-L1 (**1** is the substrate Cys-MMP-2-aPD-L1, **2** and **3** are enzymatic products). HPLC (**E**) and MS (**F**) of Cys-MP9-MMP-2-aPD-L1 (**1** is the substrate Cys-MP9-MMP-2-aPD-L1, **2** and **3** are enzymatic products).

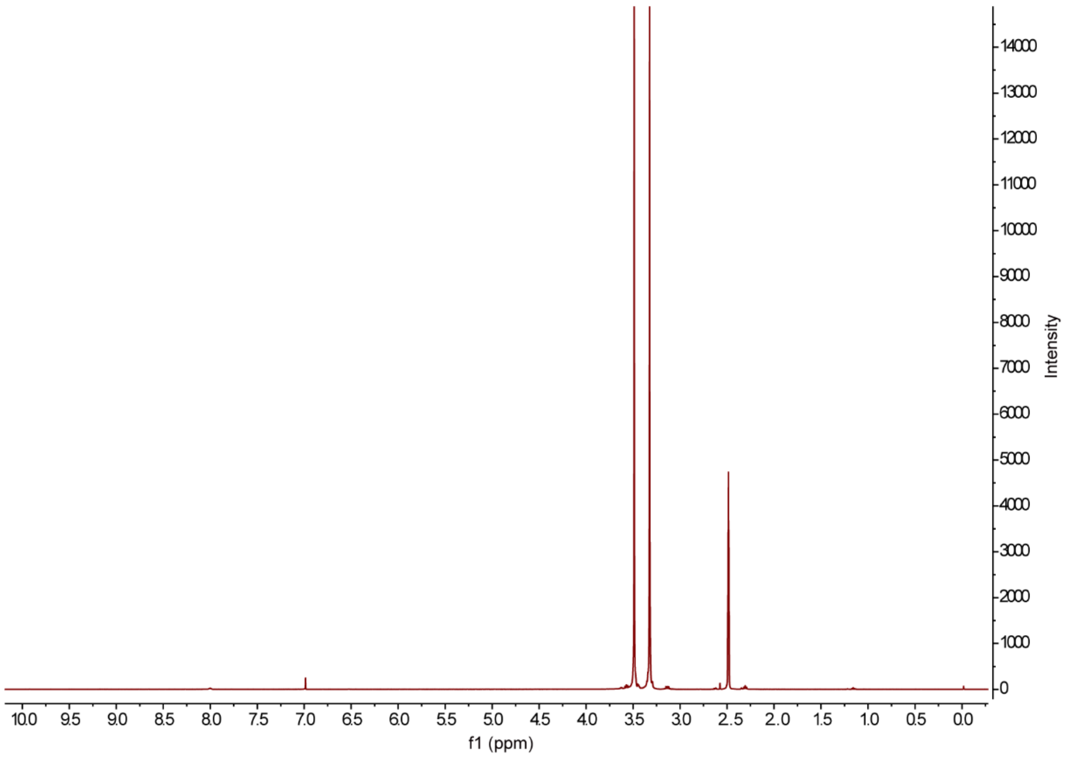

Figure S4. ^{1}H NMR of compound 4-arm PEG-Mal.

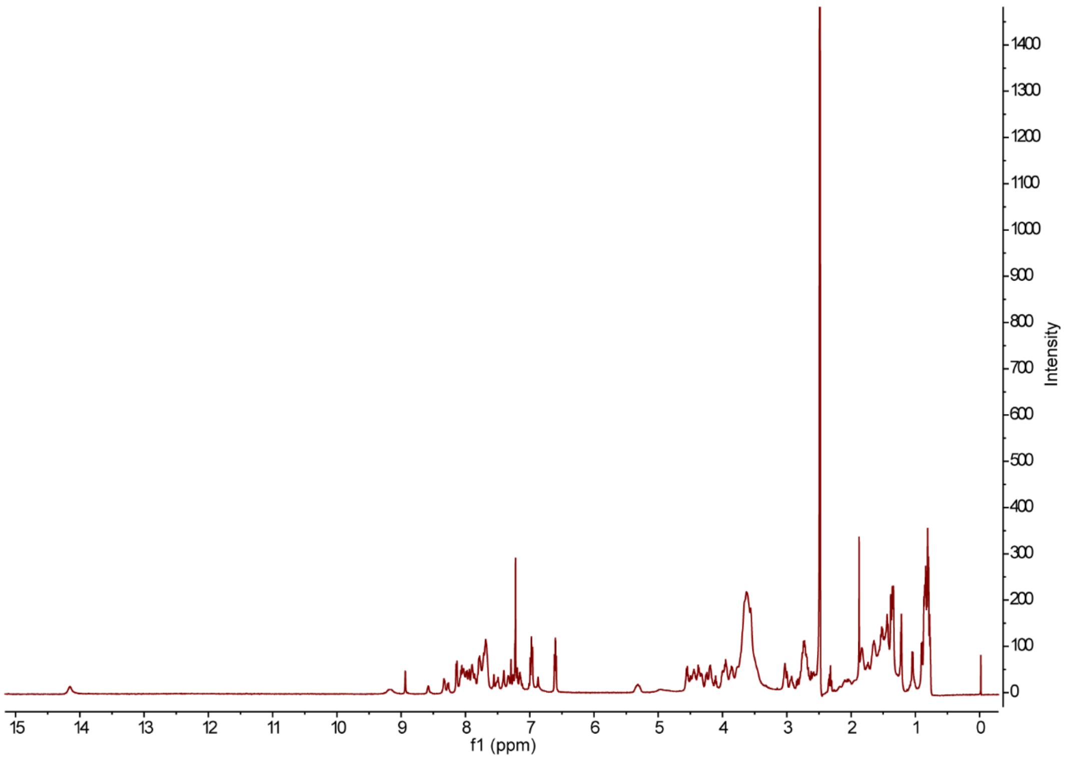

Figure S5 . ^{1}H NMR of compound Cys-MP9-MMP-2-aPD-L1.

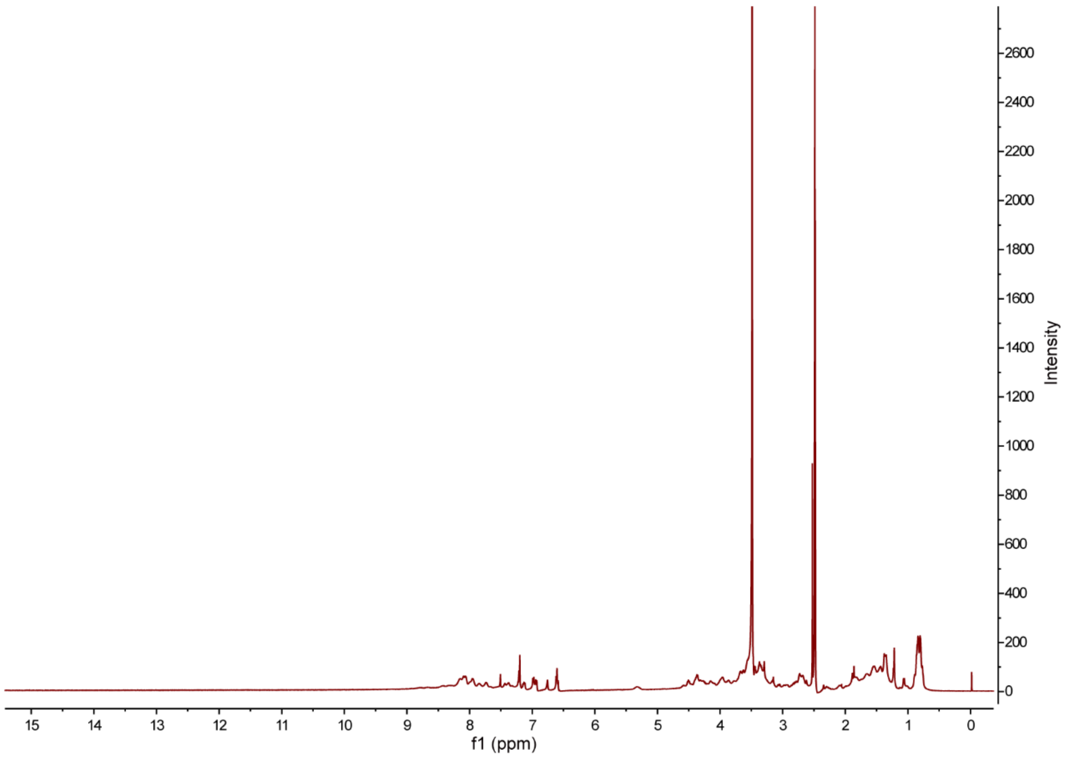

Figure S6. ^{1}H NMR of compound PEG-MP9-aPDL1.

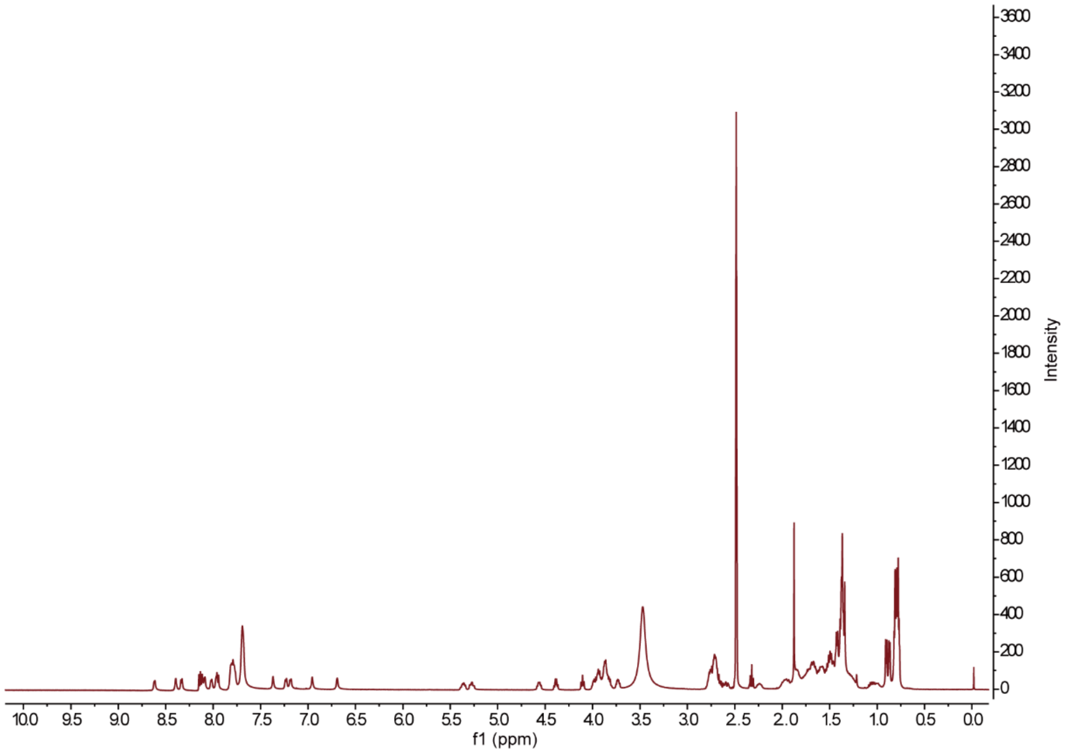

Figure S7. ^{1}H NMR of compound Cys-MP9.

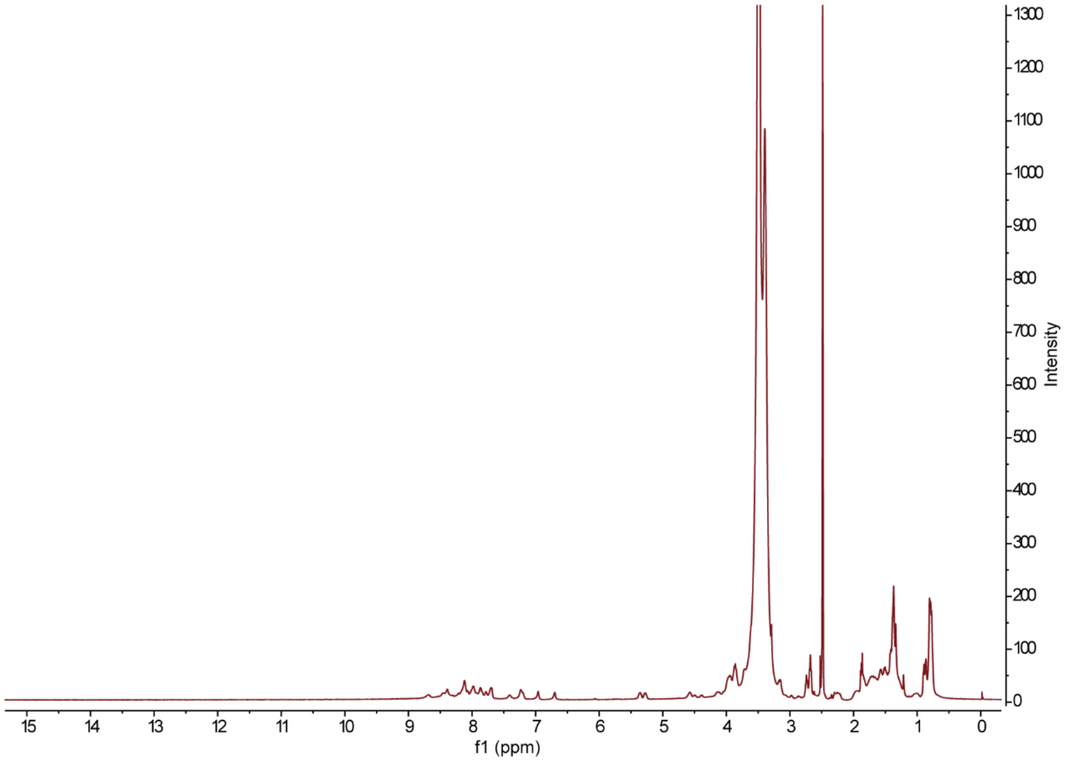

Figure S8. ^{1}H NMR of compound PEG-MP9.

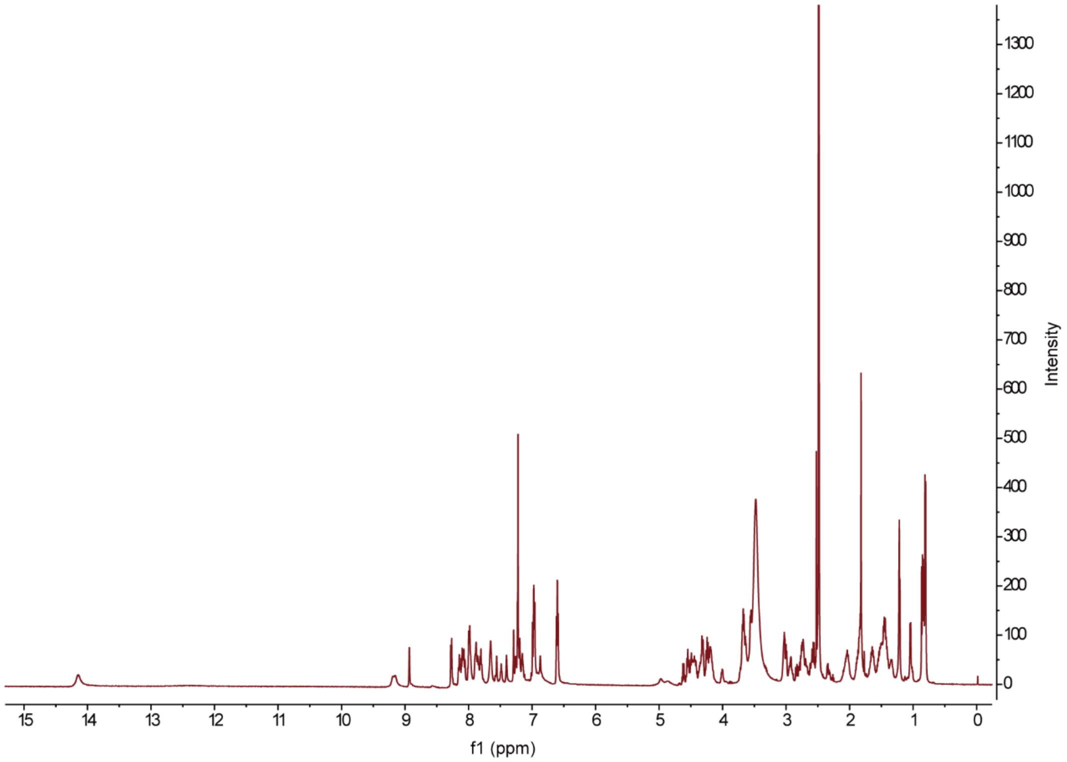

Figure S9. ^{1}H NMR of compound Cys-MMP-2-aPD-L1.

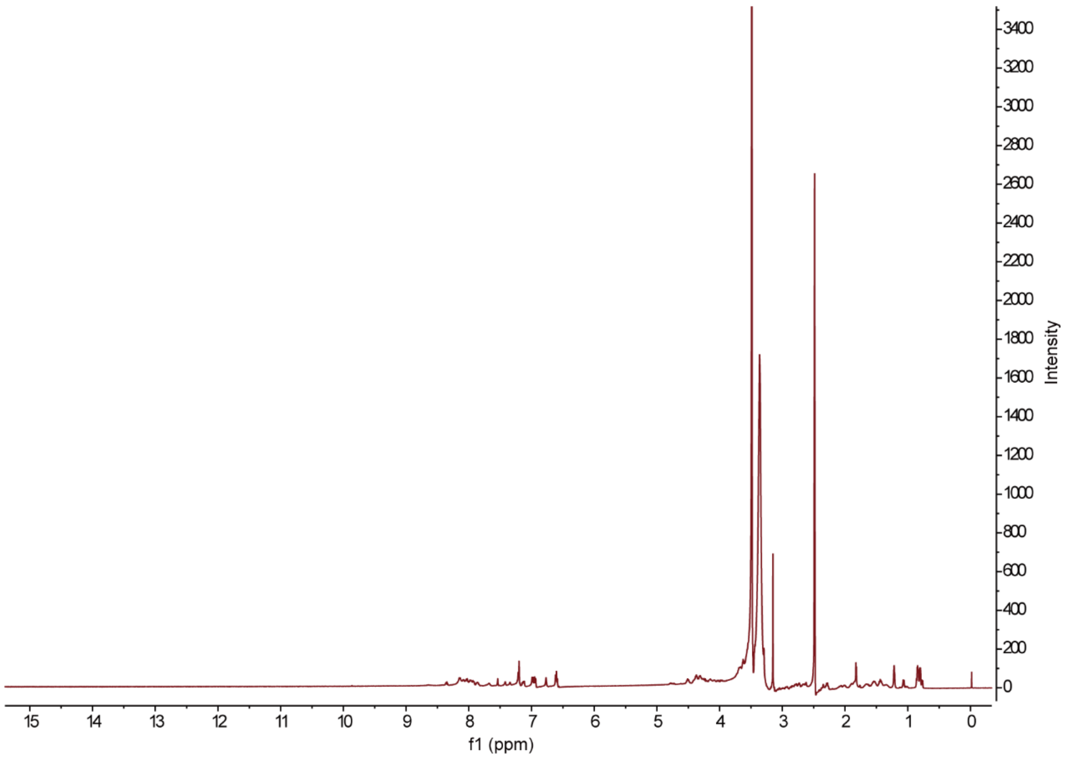

Figure S10. ^1H NMR of compound PEG-aPDL1.

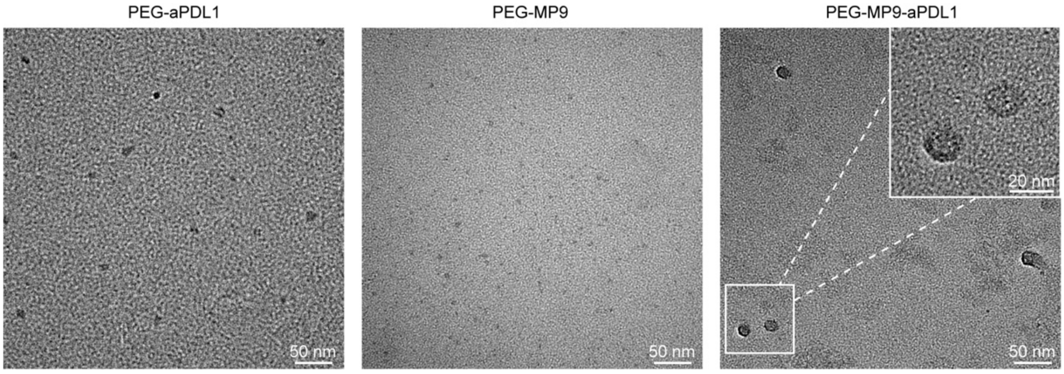

Figure S11. The transmission electron microscopy of PEG-aPDL1, PEG-MP9, and PEG-MP9-aPDL1.

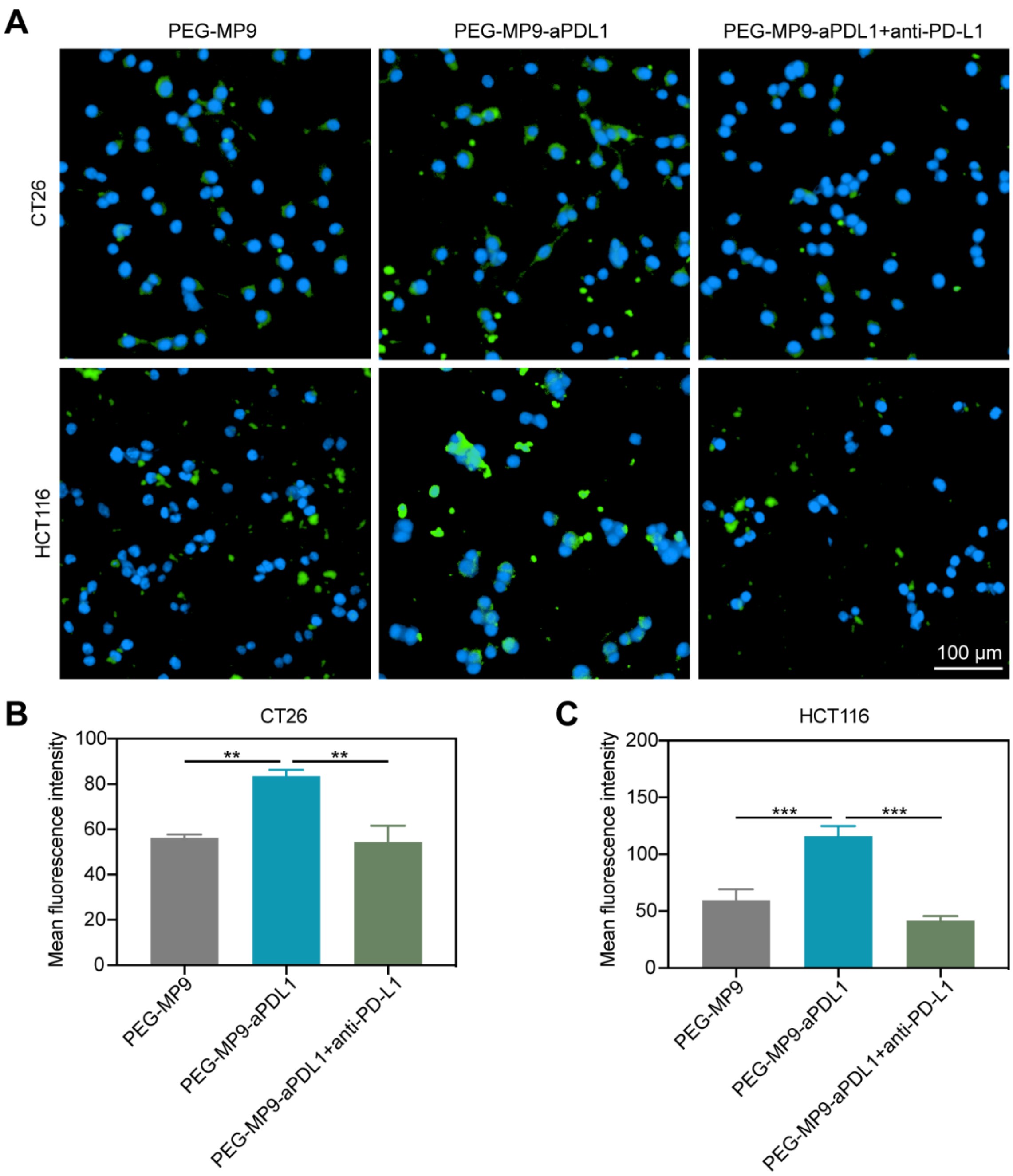

Figure S12. (**A**) High content system images showed cell uptake after 30 min of FITC-PEG-MP9 and FITC-PEG-MP9-aPDL1 with or without free anti-PD-L1. The mean fluorescence intensity in CT26 (**B**) and HCT116 (**C**) after incubation in PEG-MP9, and PEG-MP9-aPDL1 for 30 min with or without free anti-PD-L1 peptide. Data are presented as mean ± s.d.; n = 3. Statistical significance: $^{**}p < 0.01$, and $^{***}p < 0.001$.

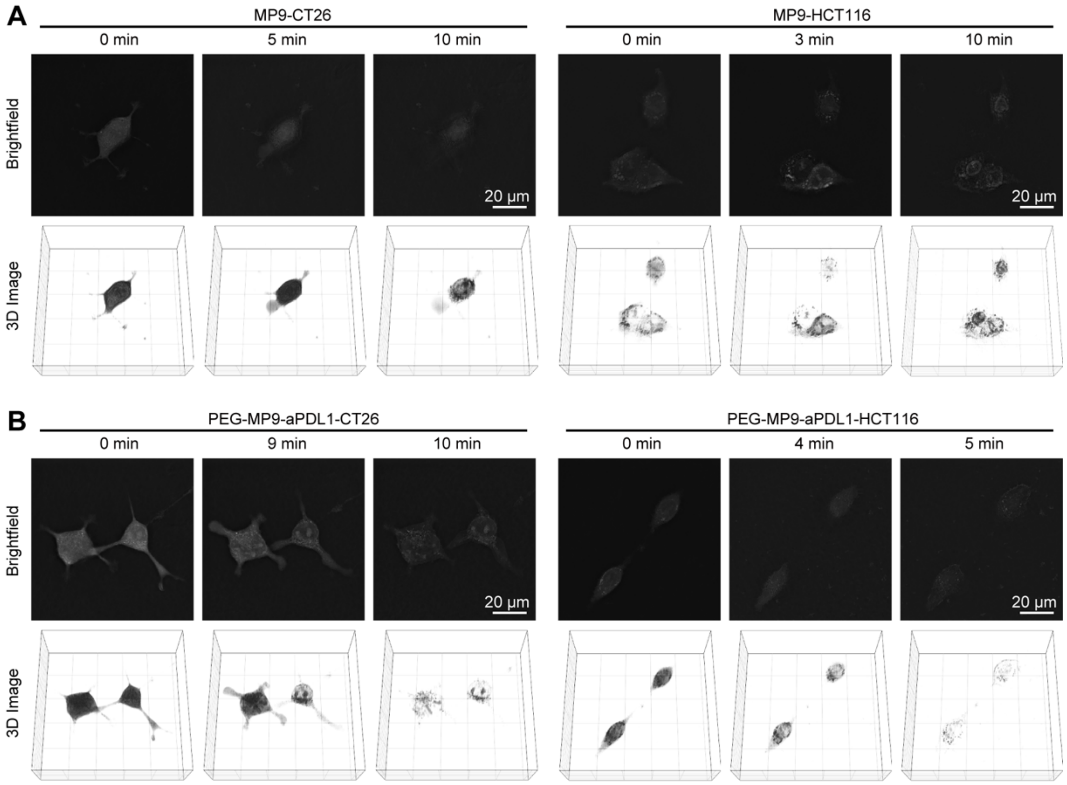

Figure S13. Brightfield microscopy and 3D images of MP9 (**A**) and PEG-MP9-aPDL1 (**B**) treated CT26 and HCT116.

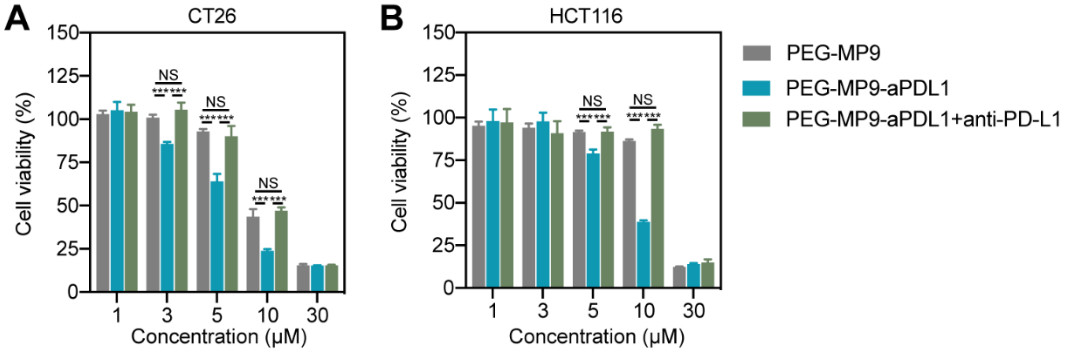

Figure S14. Cell viability analysis of CT26 (**A**) and HCT116 (**B**) cultured in PEG-MP9, and PEG-MP9-aPDL1 with or without free anti-PD-L1. Data are presented as mean ± s.d.; n = 3. Statistical significance: $^{***}p < 0.001$.

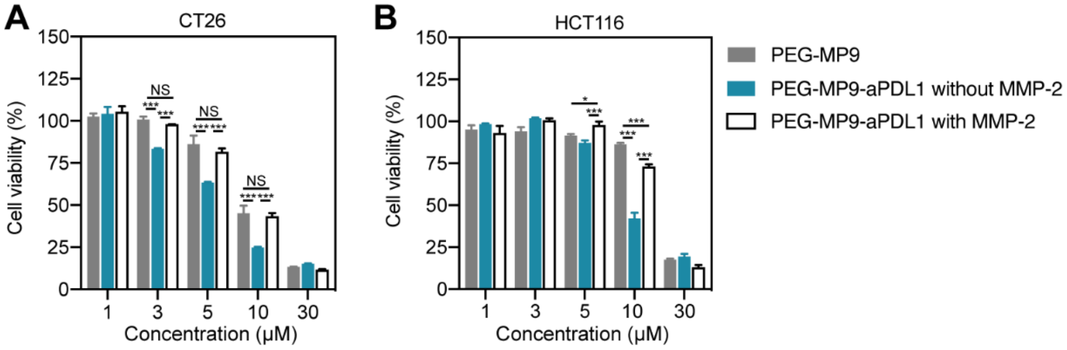

Figure S15. Cell viability analysis of CT26 (**A**) and HCT116 (**B**) cultured in PEG-MP9, and PEG-MP9-aPDL1 with or without exogenous MMP-2. Data are presented as mean ± s.d.; n = 3. Statistical significance: *$p < 0.05$, and ***$p < 0.001$.

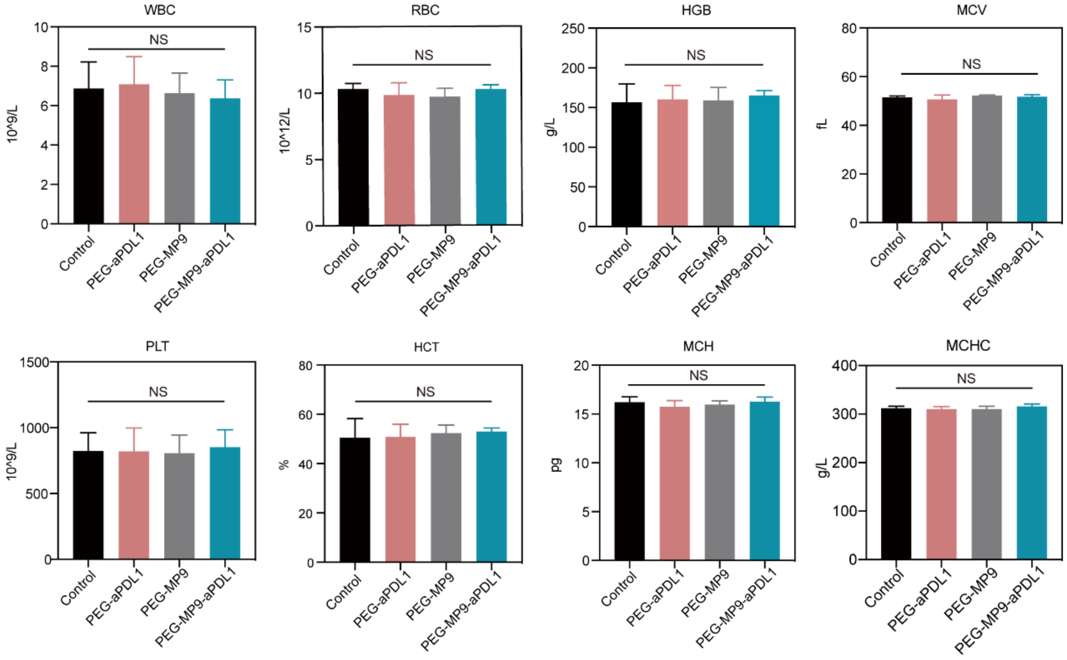

Figure S16. The blood samples were collected in each group corresponding to **Figure 6B** and analyzed to determine the hematological toxicity. White blood cells (WBC), red blood cells (RBC), hemoglobin (HGB), mean corpuscular volume (MCV), platelet (PLT), hematocrit (HCT), mean corpuscular hemoglobin (MCH), and mean corpuscular hemoglobin concentration (MCHC). Data are presented as mean ± s.d.; n = 6.

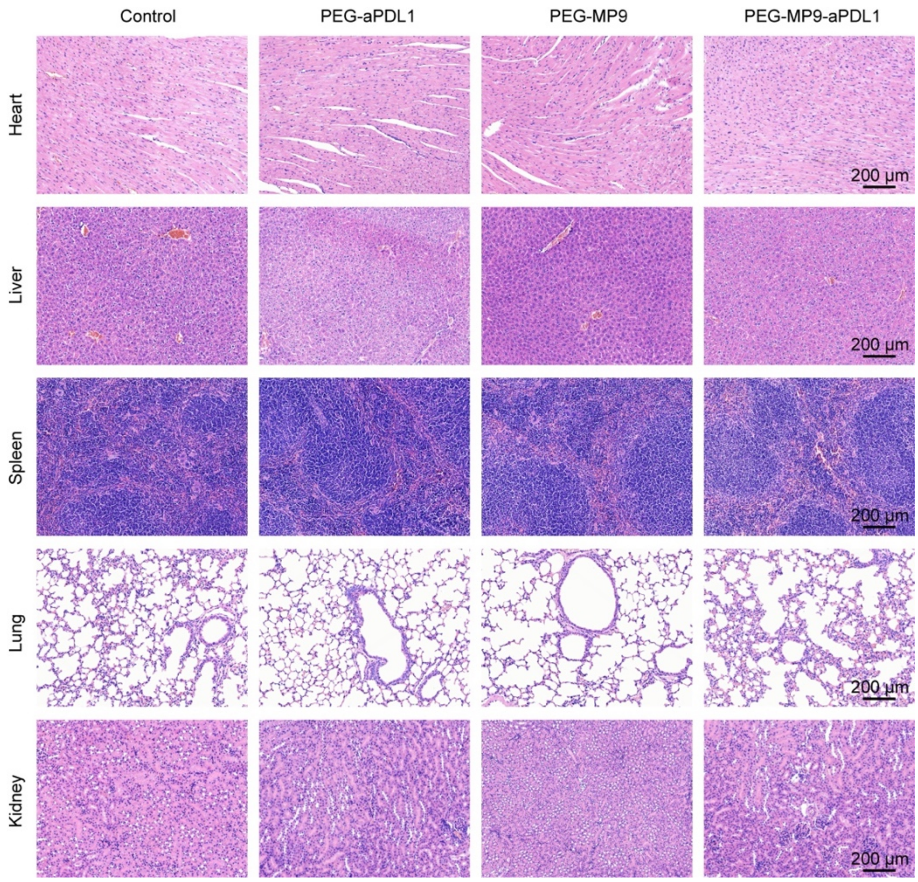

Figure S17. HE staining images of main organs (heart, liver, spleen, lung, kidney) obtained from mice bearing CT26 subcutaneous tumors in each group corresponding to **Figure 6B**.

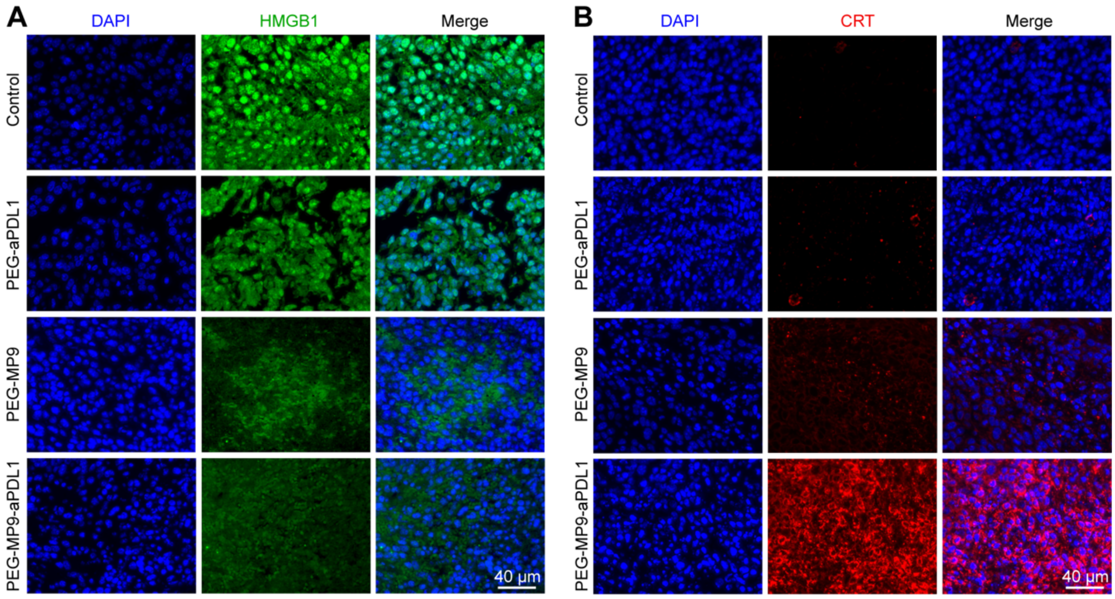

Figure S18. HMGB1 and CRT expression of tumors in each group corresponding to **Figure 6B**.

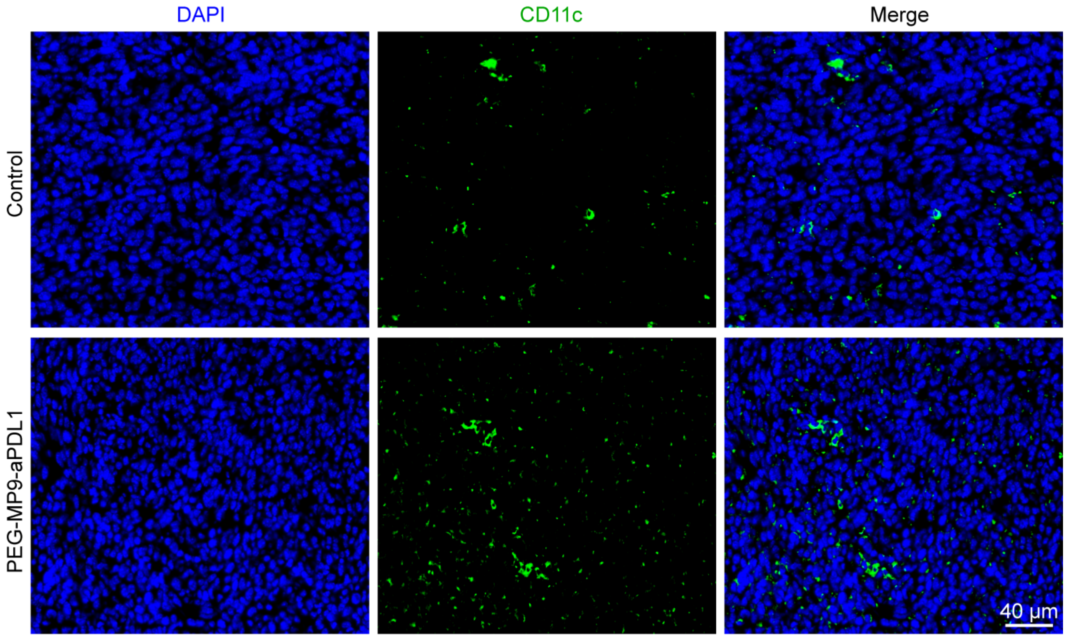

Figure S19. CD11c expression of tumors in Control and PEG-MP9-aPDL1 groups corresponding to **Figure 6B**.

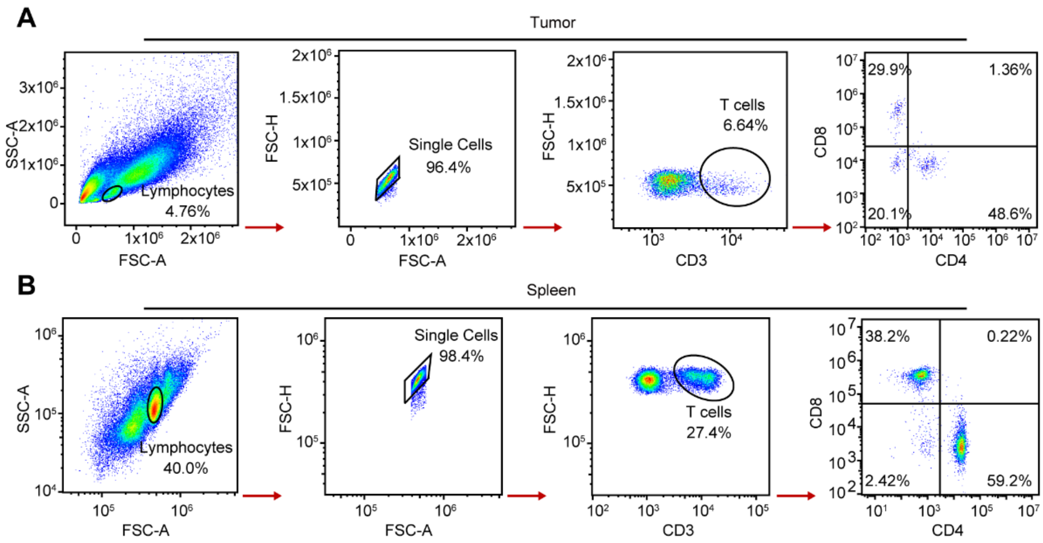

Figure S20. The strategy of gating CD4+, CD8+ T cells in the tumors (A) and spleens (B) of CT26 tumor bearing mice by flow cytometry analysis after PBS, PEG-aPDL1, PEG-MP9, and PEG-MP9-aPDL1 treatment.

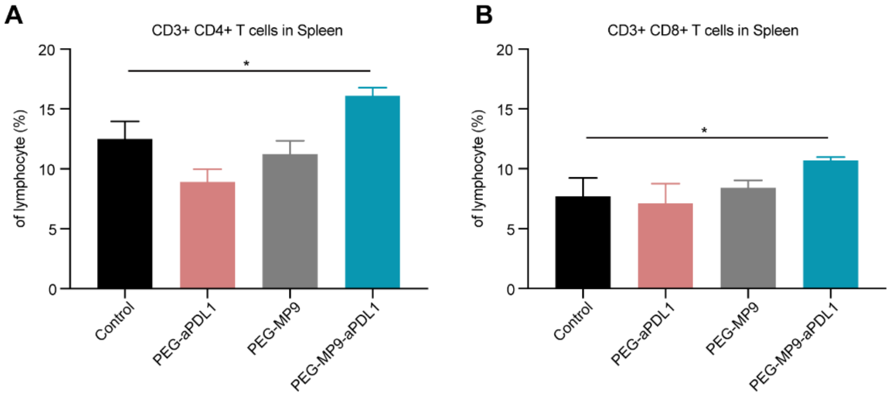

Figure S21. Flow cytometry analysis of CD4+, CD8+ T cells isolated from spleens of CT26 tumor bearing mice after PBS, PEG-aPDL1, PEG-MP9, and PEG-MP9-aPDL1 treatment. Data are presented as mean ± s.d.; n = 3. Statistical significance: *$p < 0.05$.

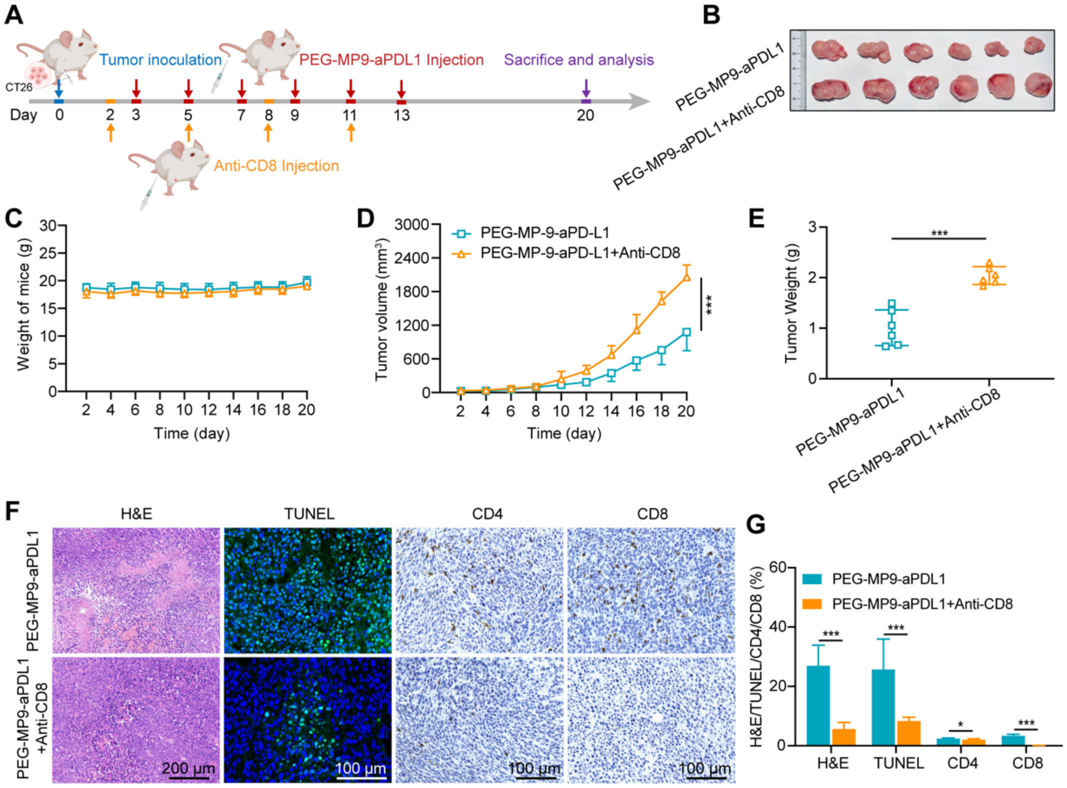

Figure S22. (**A**) Schematic illustration of the timeline for the CD8+ T cells depletion experiment *in vivo*. (**B**) Images of excised CT26 tumors. (**C**) Body weight of mice bearing CT26 tumors throughout the study. (**D**) Tumor growth curves. (**E**) Weight of CT26 tumors. Representative immunohistochemical staining images of HE, TUNEL, CD4, and CD8 of the tumor sections in different groups (**F**) and quantified results (**G**). Data are presented as mean ± s.d.; n = 6. Statistical significance: *$p < 0.05$, and ***$p < 0.001$.

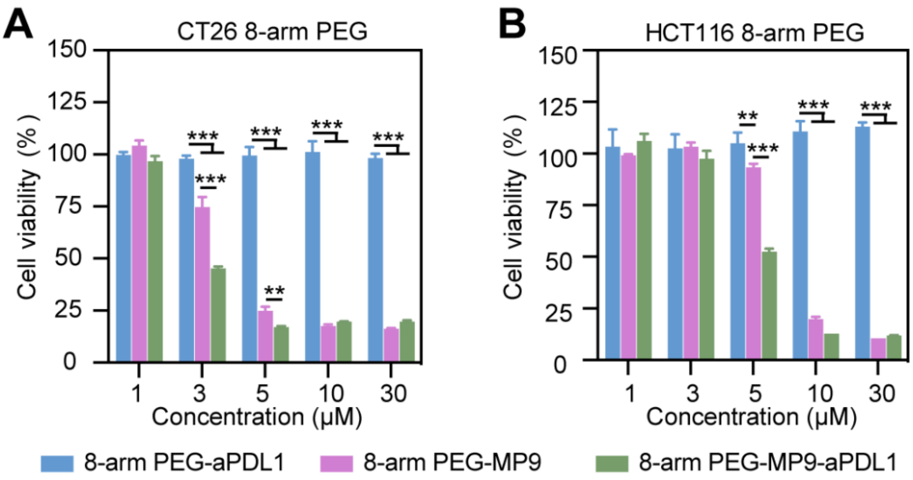

Figure S23. Cell viability analysis of CT26 and HCT116 cells cultured in 8-arm PEG-aPDL1, 8-arm PEG-MP9, and 8-arm PEG-MP9-aPDL1. Data are presented as mean ± s.d.; n = 3. Statistical significance: **$p < 0.01$, and ***$p < 0.001$.

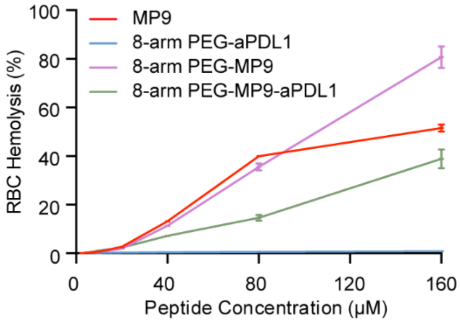

Figure S24. RBC hemolytic activity of MP9, 8-arm PEG-aPDL1, 8-arm PEG-MP9, and 8-arm PEG-MP9-aPDL1. Data are presented as mean ± s.d.; n = 3.

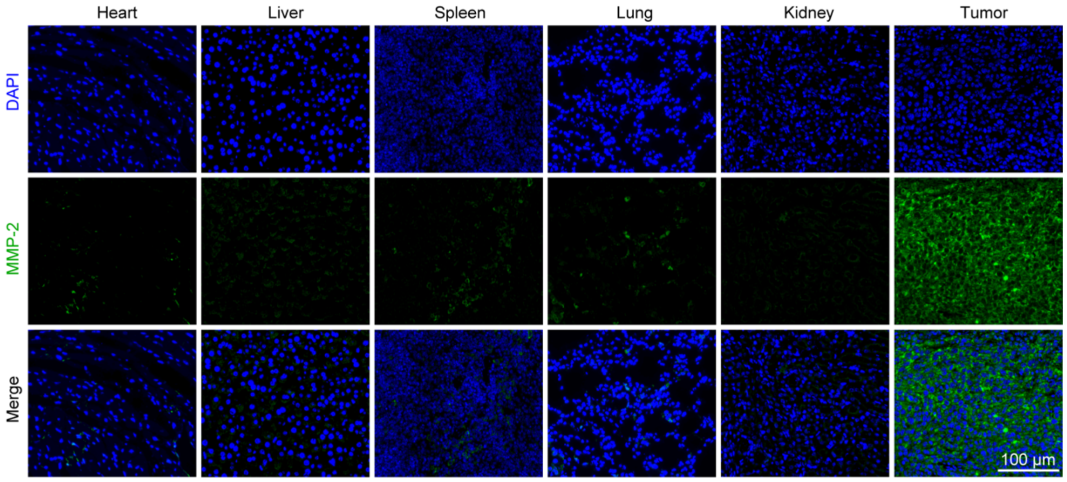

Figure S25. MMP-2 expression of heart, liver, spleen, lung, kidney, and tumor in PEG-MP9-aPDL1 group mice.

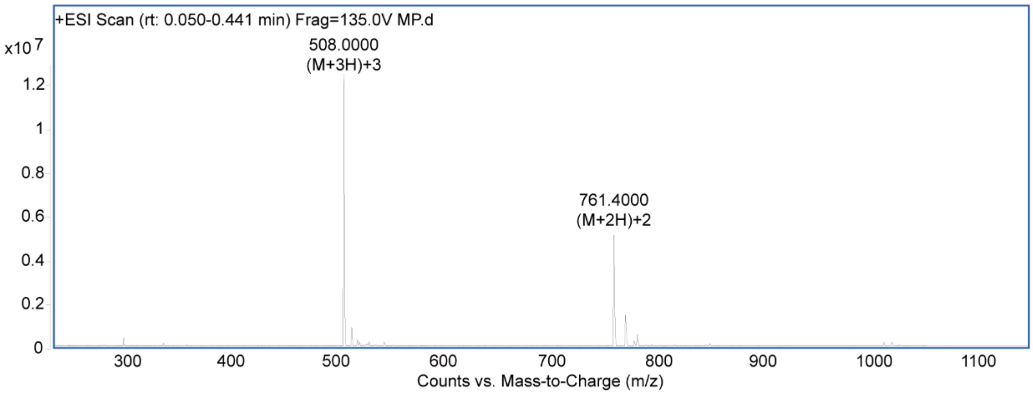

Figure S26. MS of compound MP.

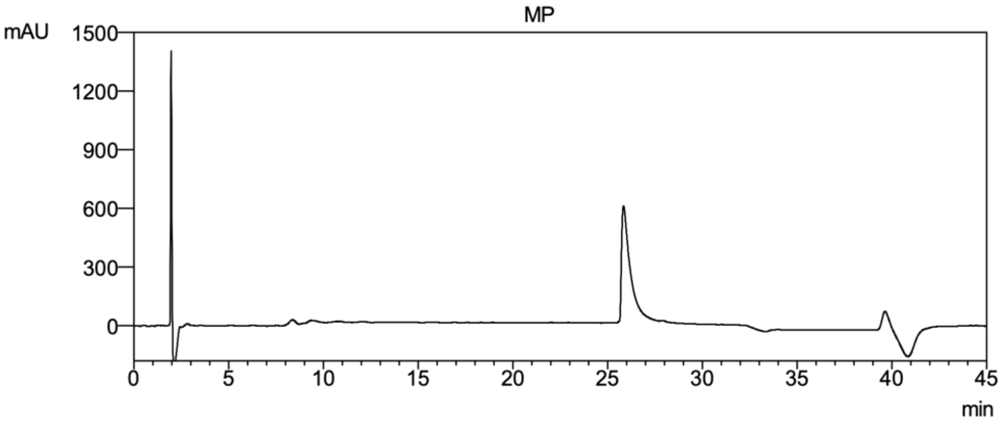

Figure S27. HPLC of compound MP.

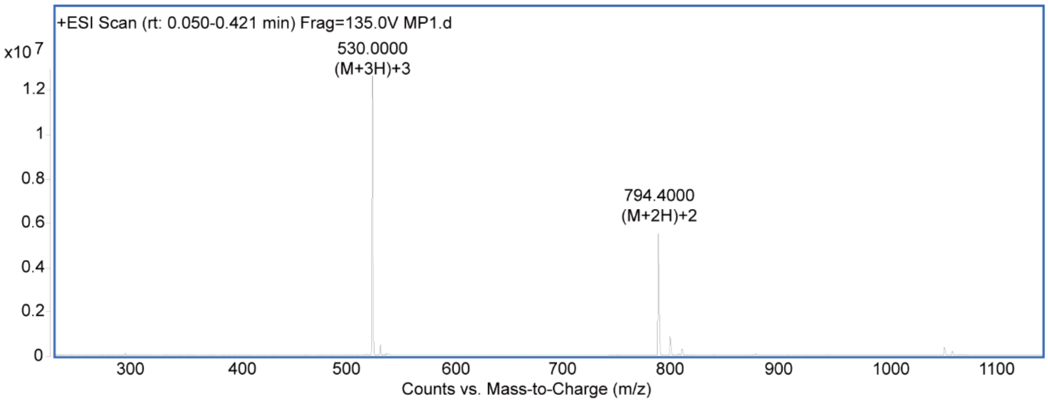

Figure S28. MS of compound MP1.

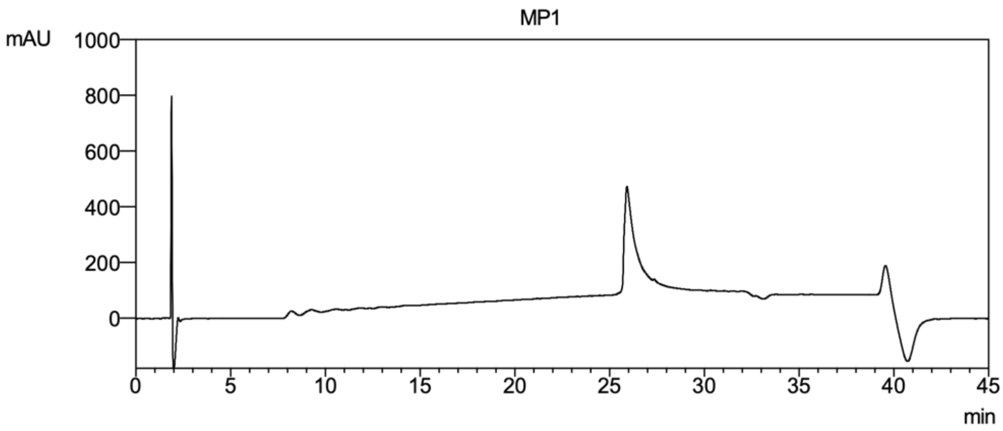

Figure S29. HPLC of compound MP1.

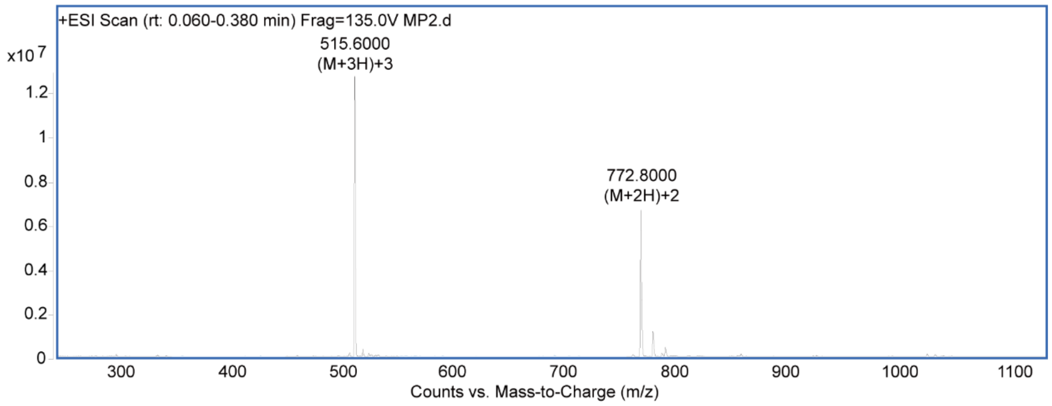

Figure S30. MS of compound MP2.

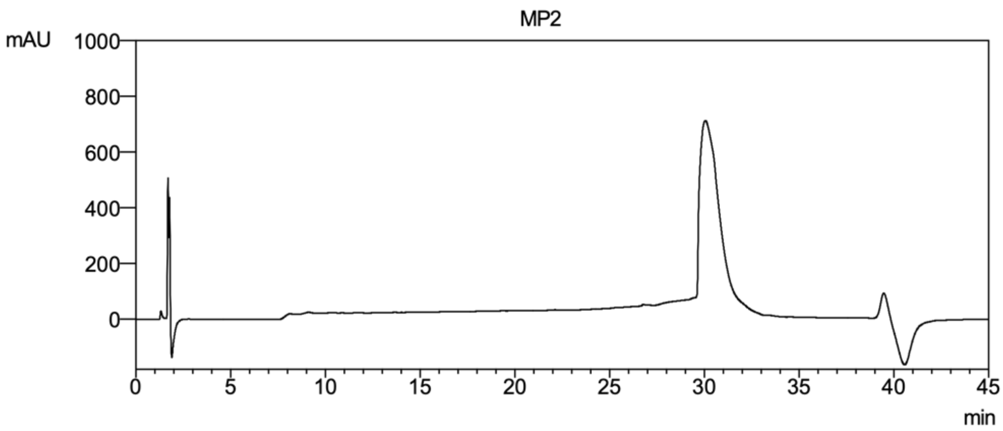

Figure S31. HPLC of compound MP2.

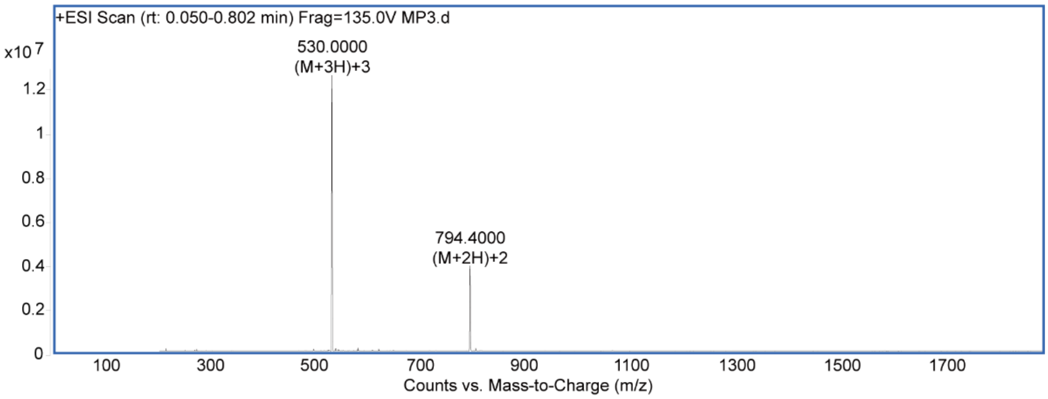

Figure S32. MS of compound MP3.

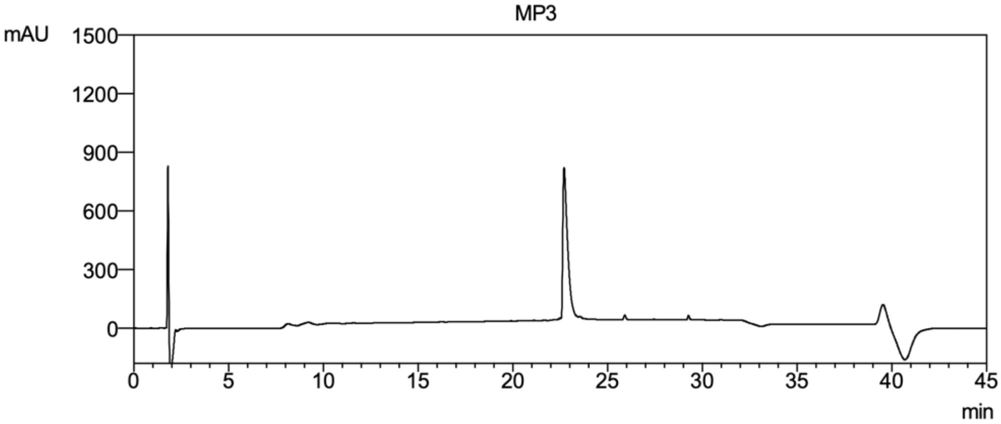

Figure S33. HPLC of compound MP3.

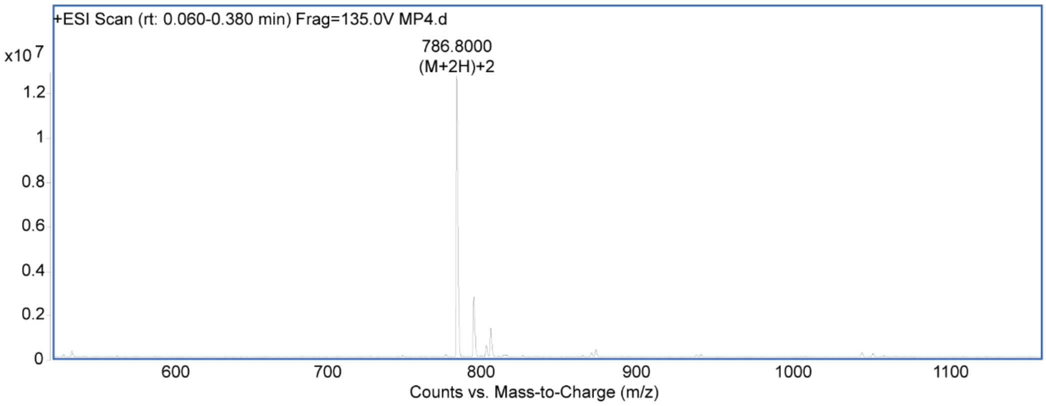

Figure S34. MS of compound MP4.

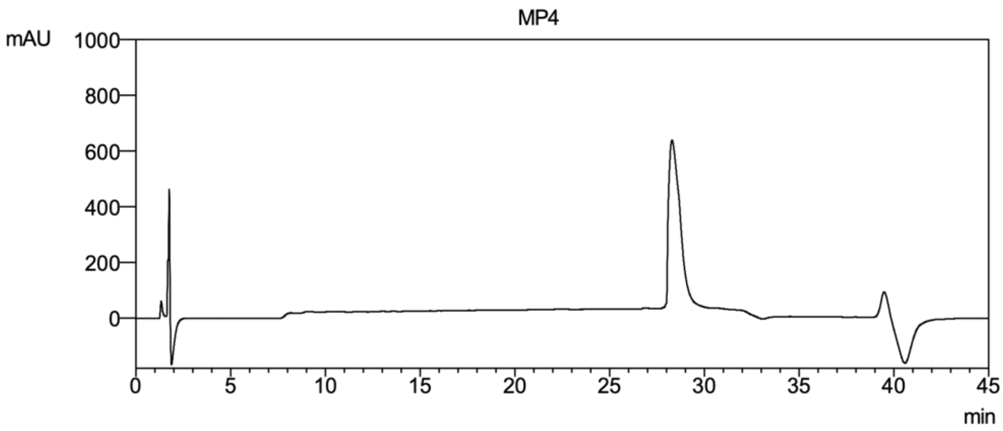

Figure S35. HPLC of compound MP4.

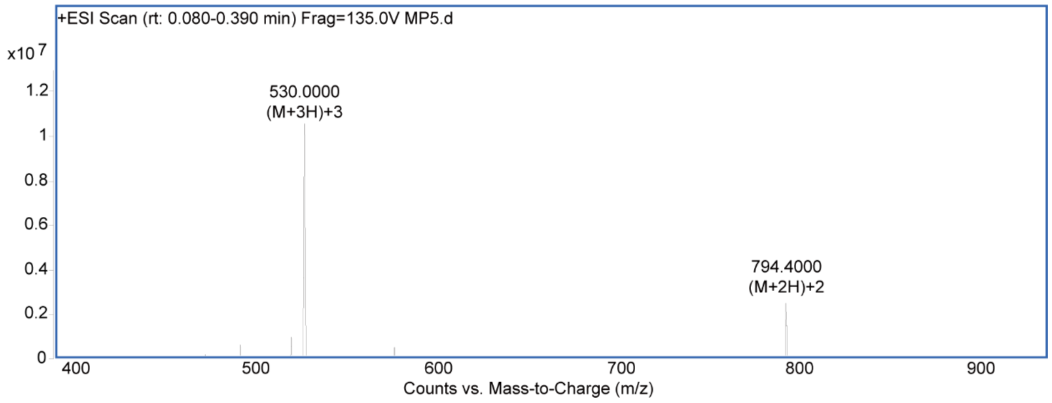

Figure S36. MS of compound MP5.

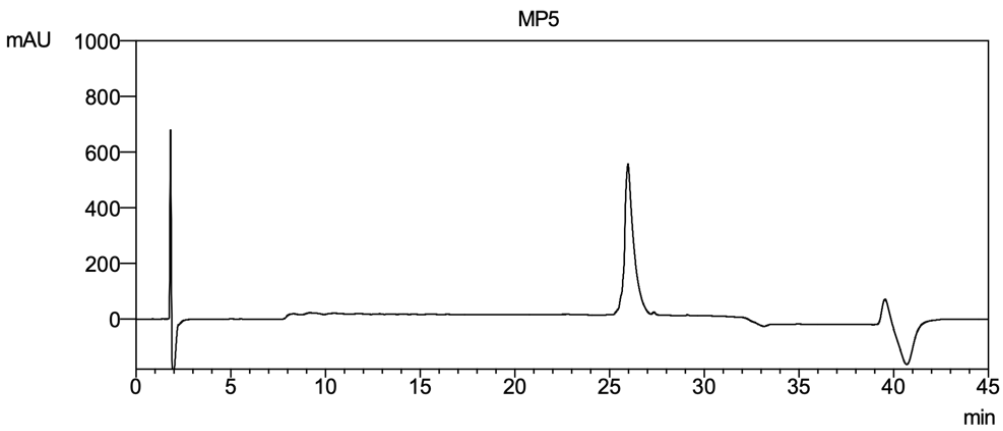

Figure S37. HPLC of compound MP5.

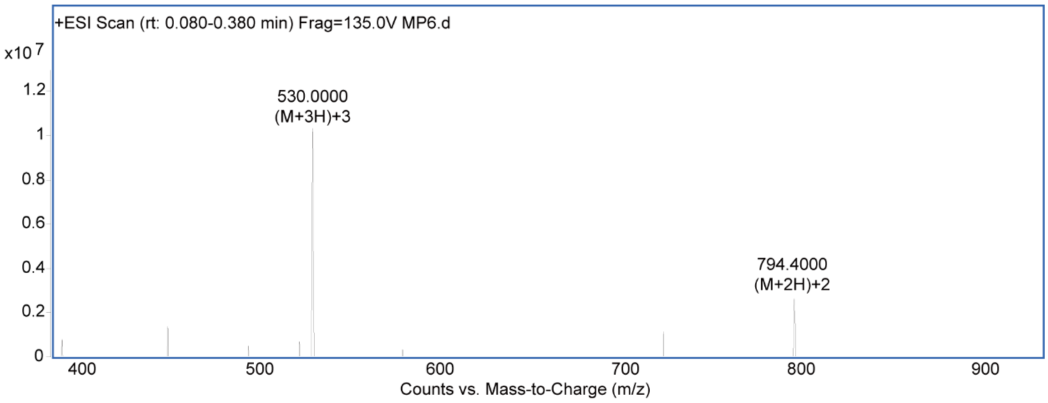

Figure S38. MS of compound MP6.

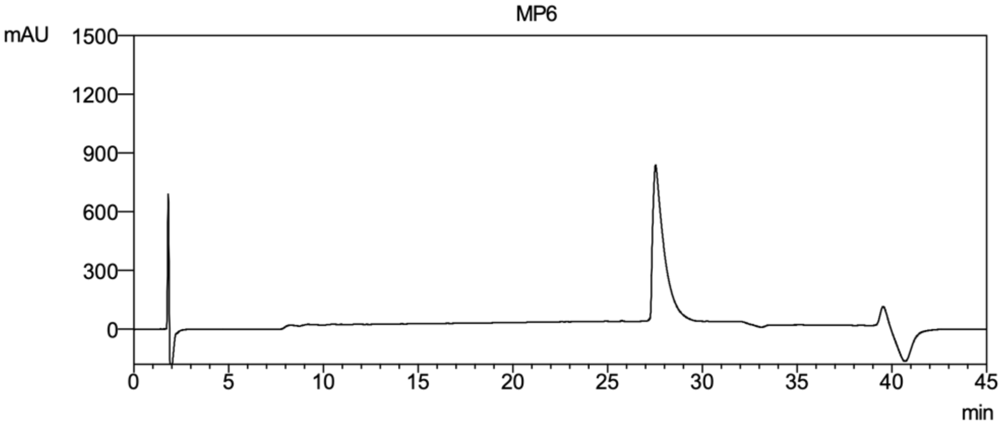

Figure S39. HPLC of compound MP6.

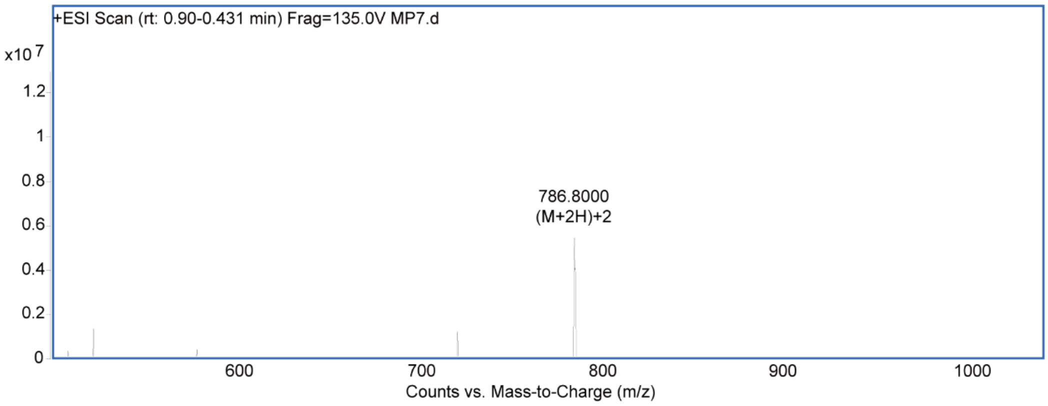

Figure S40. MS of compound MP7.

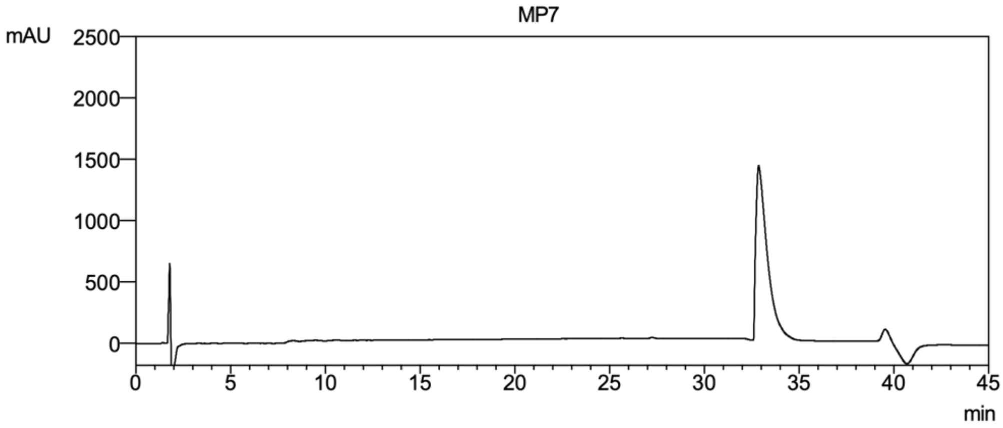

Figure S41. HPLC of compound MP7.

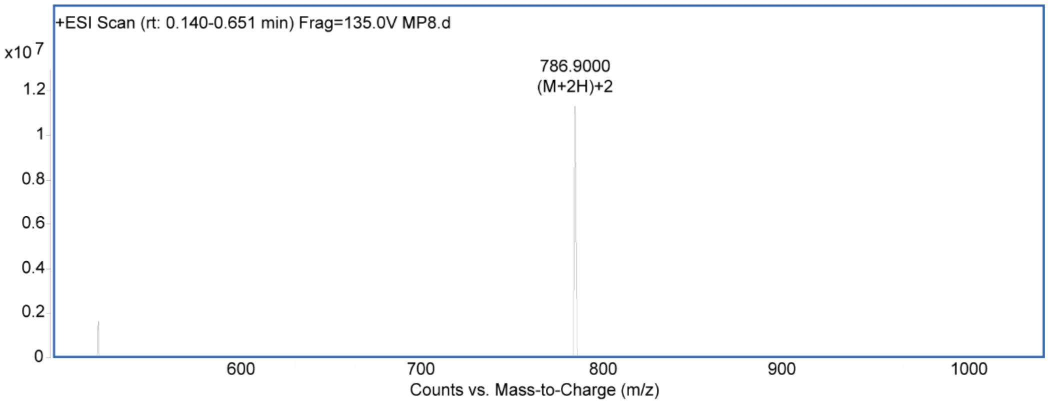

Figure S42. MS of compound MP8.

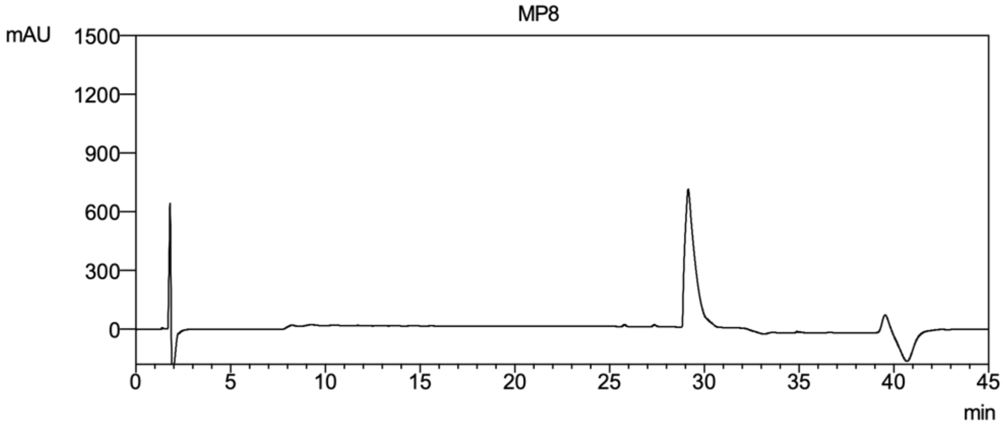

Figure S43. HPLC of compound MP8.

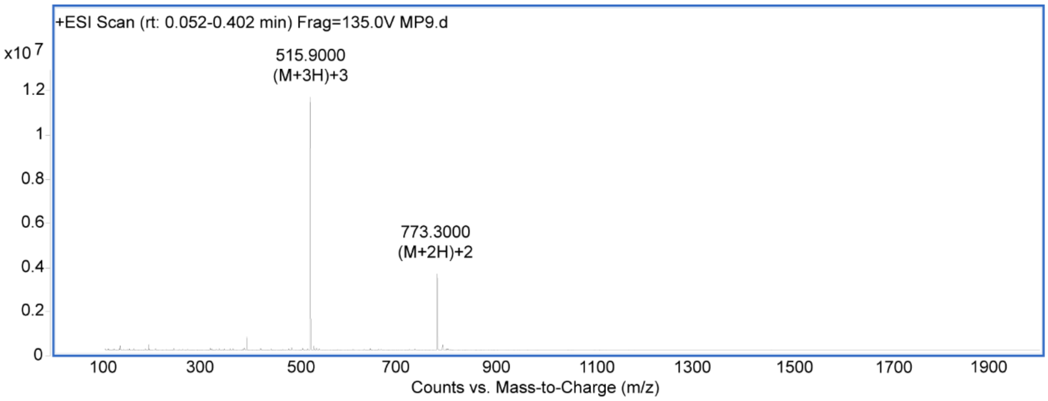

Figure S44. MS of compound MP9.

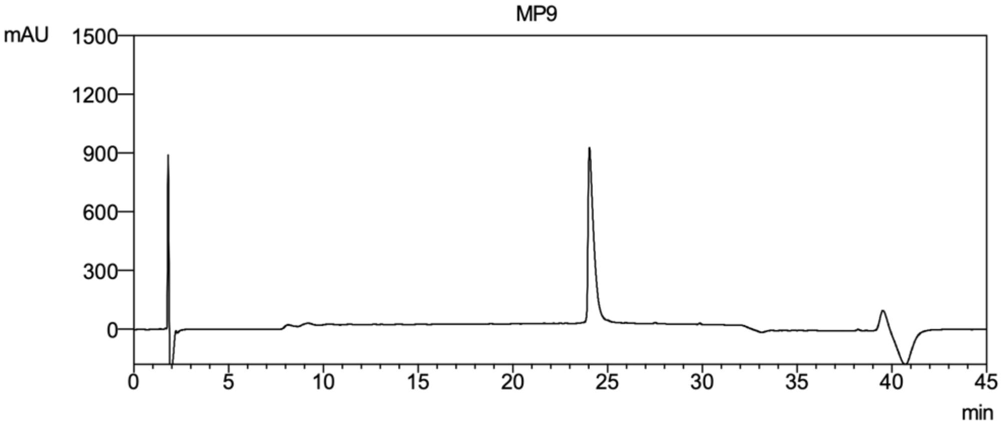

Figure S45. HPLC of compound MP9.

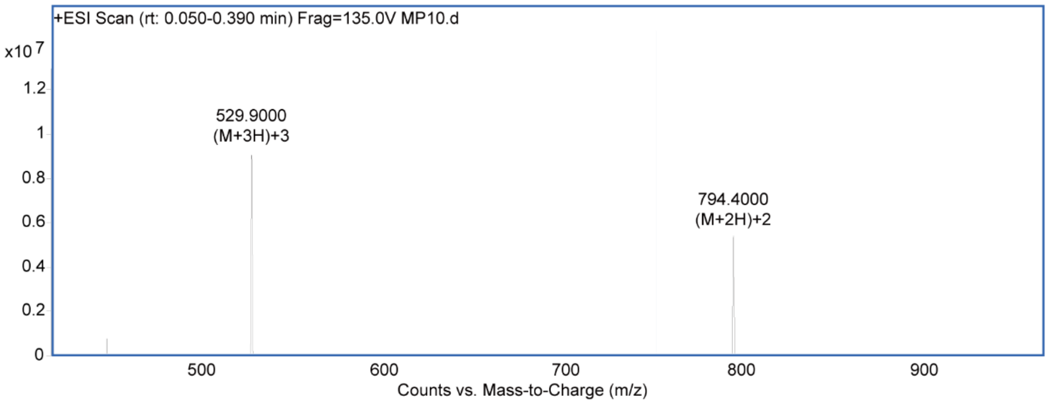

Figure S46. MS of compound MP10.

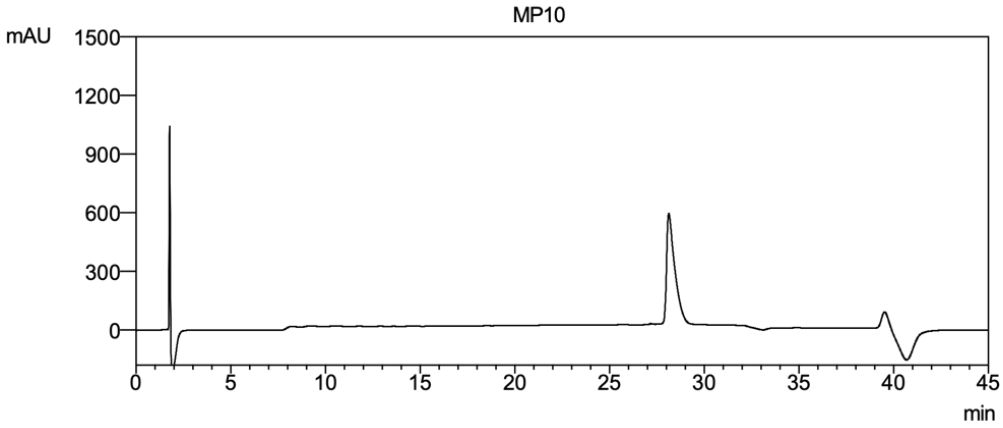

Figure S47. HPLC of compound MP10.

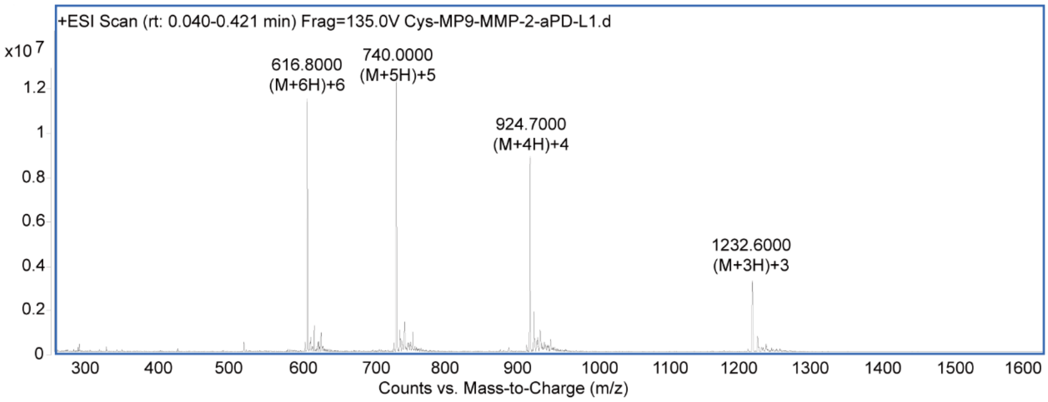

Figure S48. MS of compound Cys-MP9-MMP-2-aPD-L1.

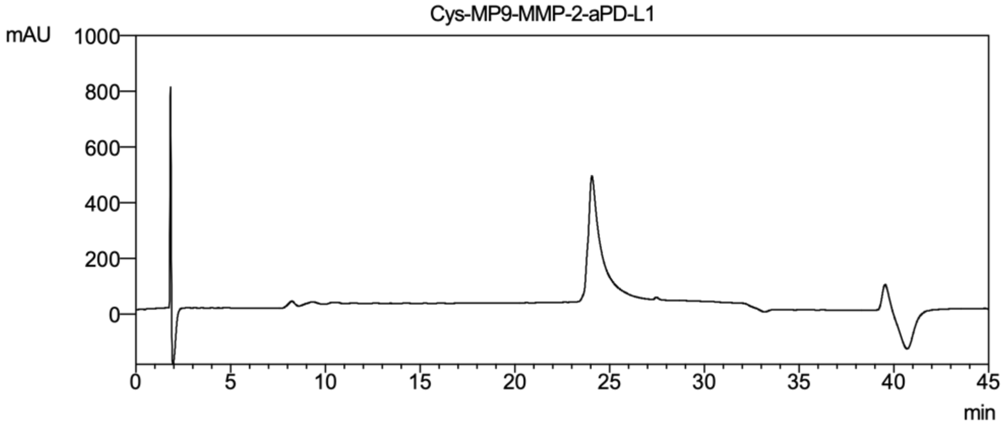

Figure S49. HPLC of compound Cys-MP9-MMP-2-aPD-L1.

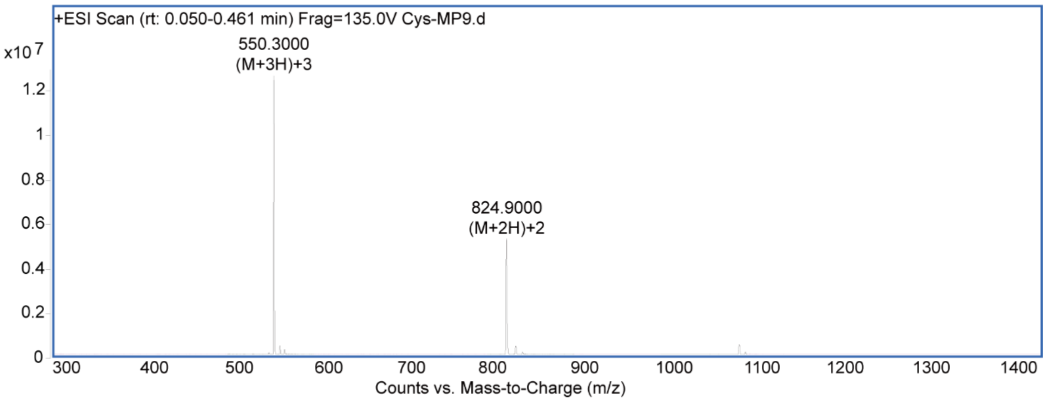

Figure S50. MS of compound Cys-MP9.

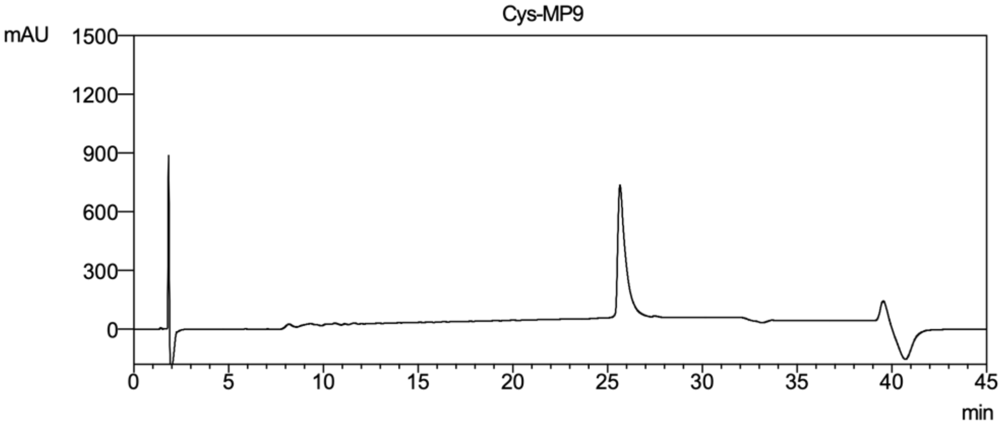

Figure S51. HPLC of compound Cys-MP9.

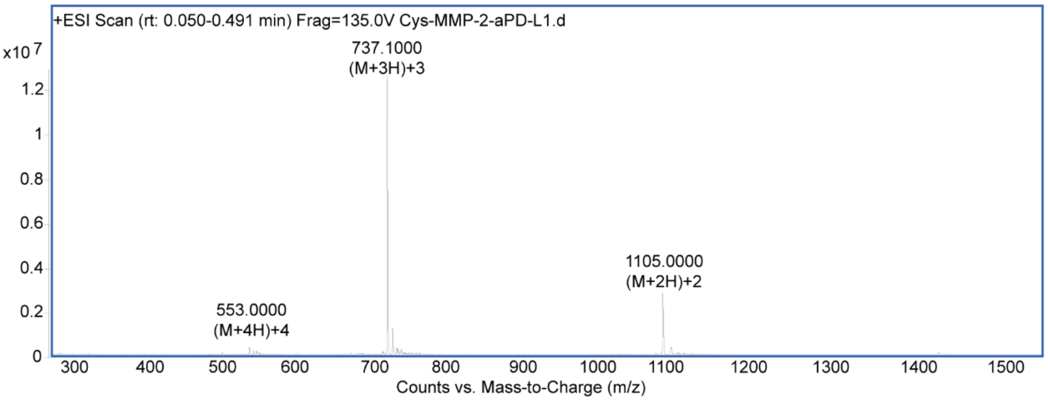

Figure S52. MS of compound Cys-MMP-2-aPD-L1.

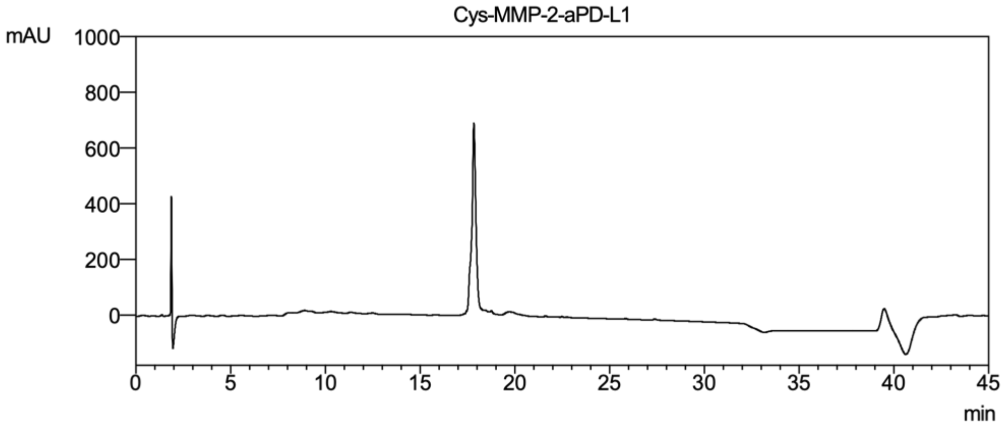

Figure S53. HPLC of compound Cys-MMP-2-aPD-L1.

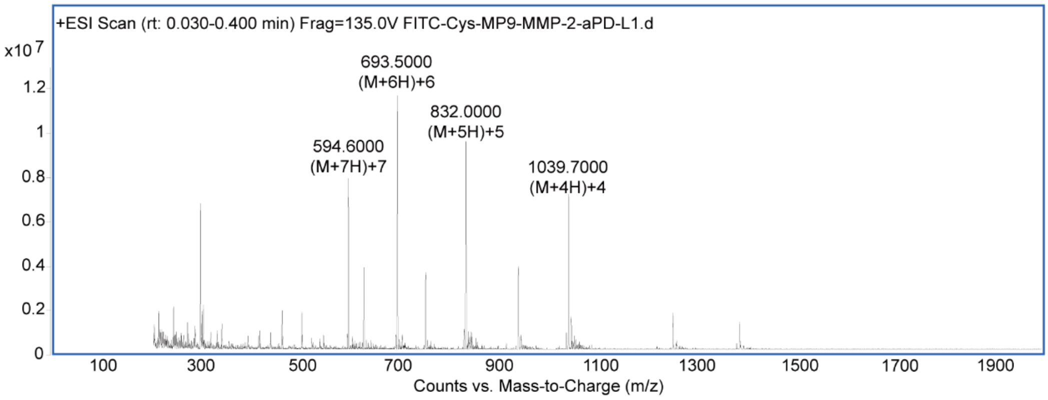

Figure S54. MS of compound FITC-Cys-MP9-MMP-2-aPD-L1.

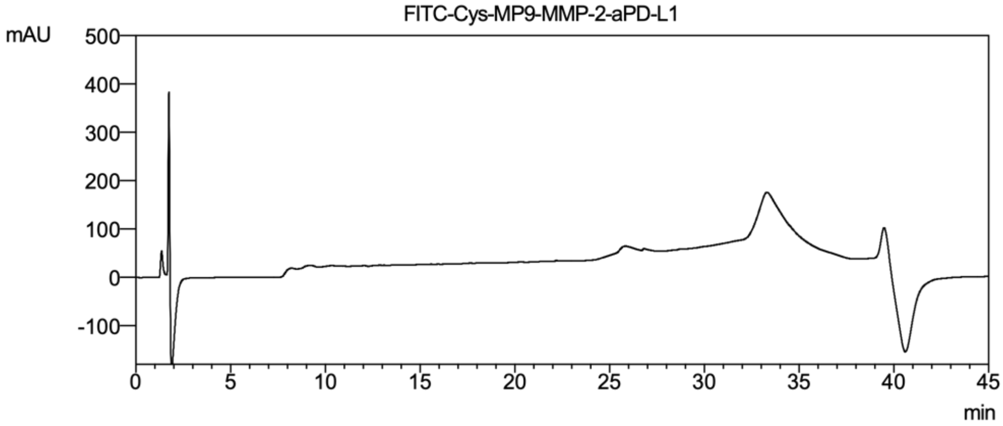

Figure S55. HPLC of compound FITC-Cys-MP9-MMP-2-aPD-L1.

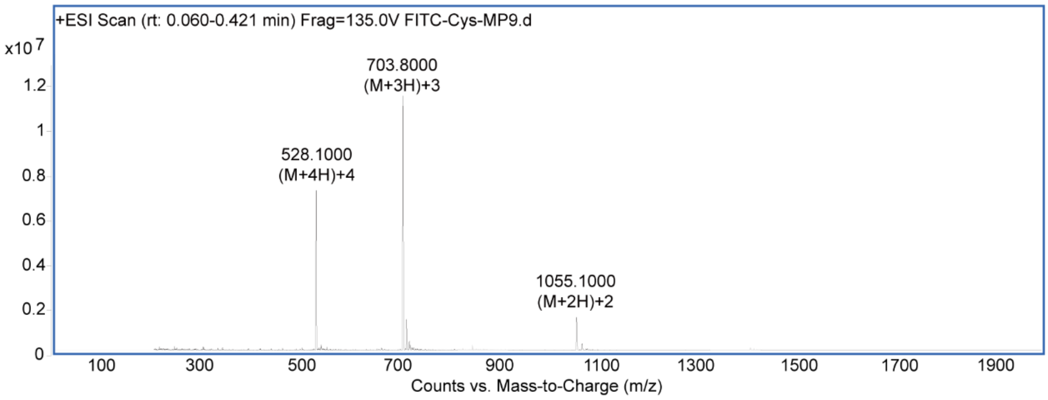

Figure S56. MS of compound FITC-Cys-MP9.

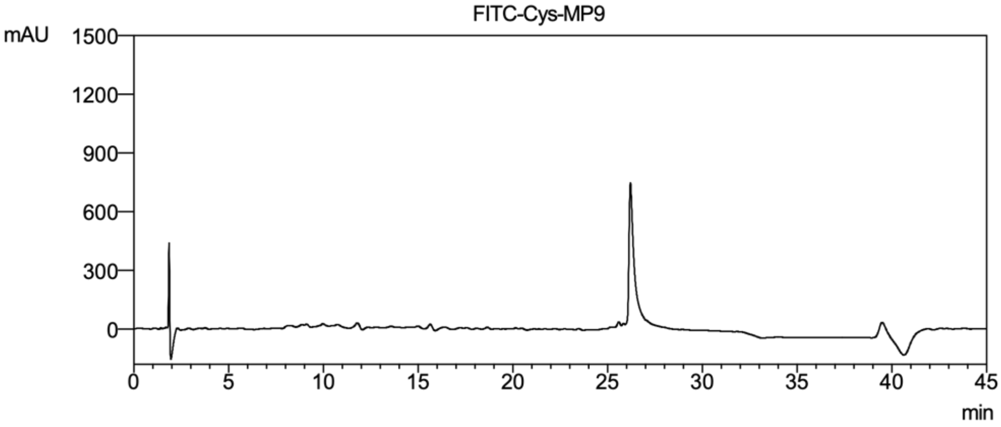

Figure S57. HPLC of compound FITC-Cys-MP9.

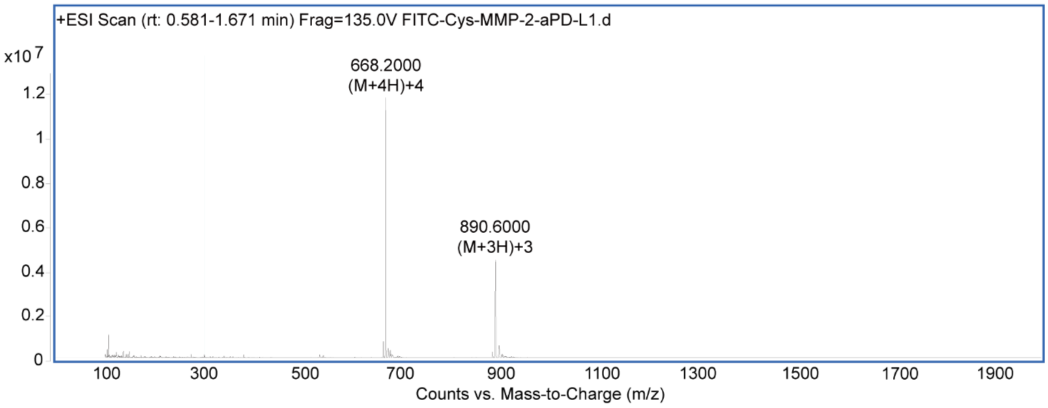

Figure S58. MS of FITC-Cys-MMP-2-aPD-L1.

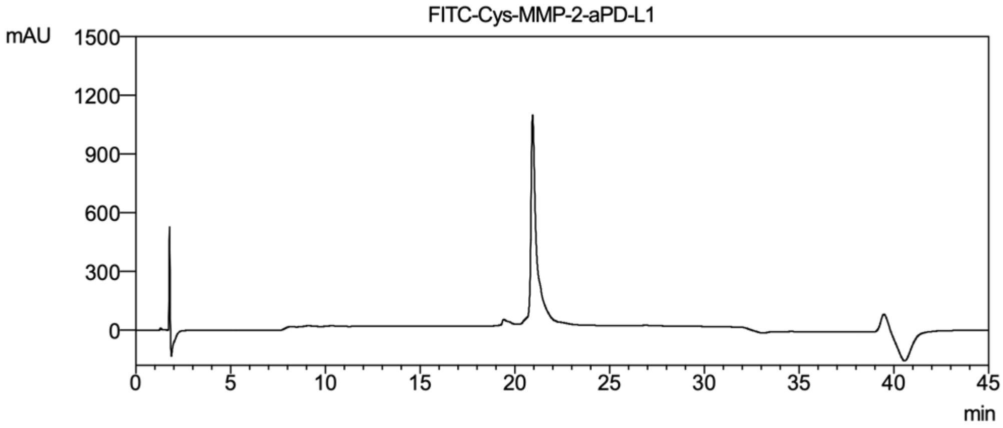

Figure S59. HPLC of compound FITC-Cys-MMP-2-aPD-L1.

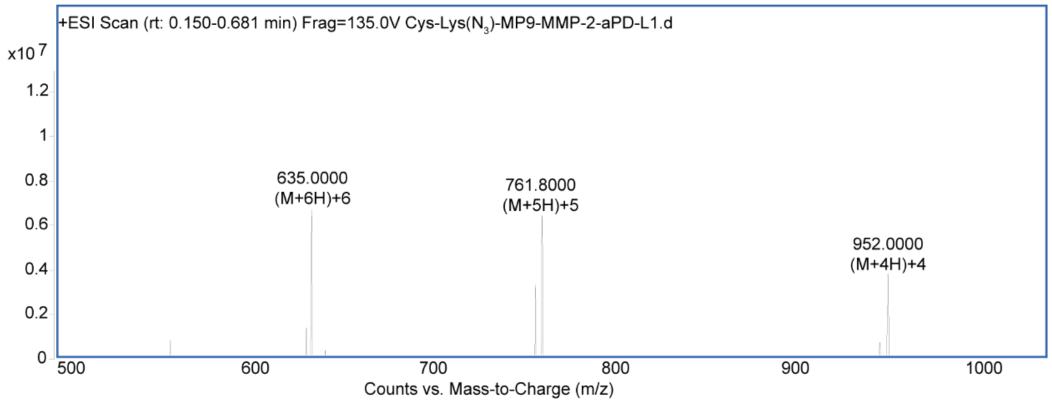

Figure S60. MS of compound Cys-Lys (N_3)-MP9-MMP-2-aPD-L1.

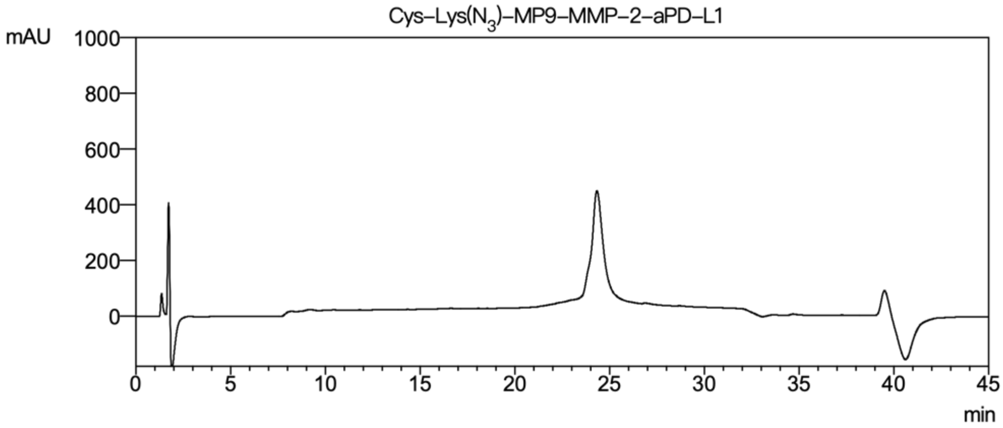

Figure S61. HPLC of compound Cys-Lys (N_3)-MP9-MMP-2-aPD-L1.

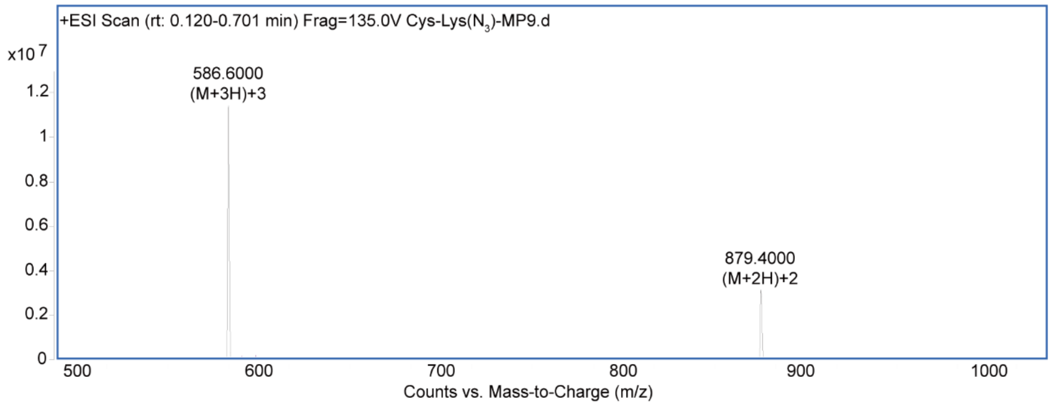

Figure S62. MS of compound Cys-Lys (N_3)-MP9.

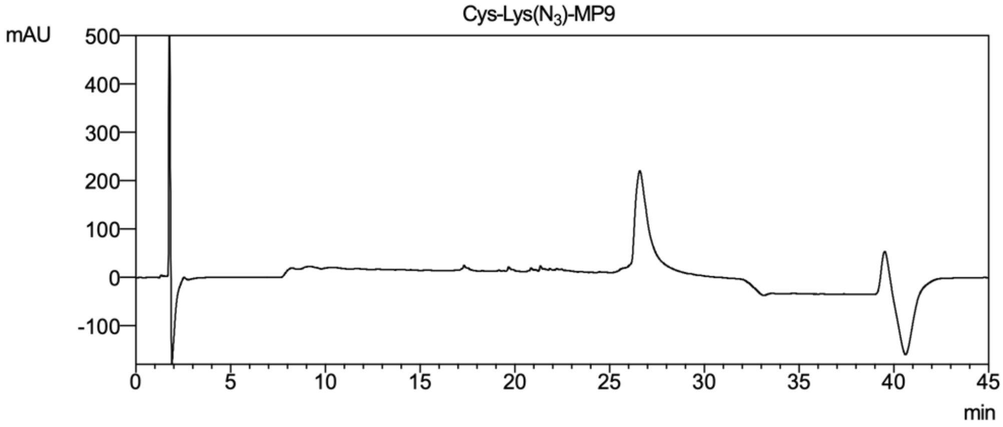

Figure S63. HPLC of compound Cys-Lys (N_3)-MP9.

Table S1. Exact mass (EM), isolated yield and electrospray MS data for peptides (positive mode).

Peptide	EM (g/mol)	Isolated yield (%)	Found mass
MP	1520.02	13.16	$[M+2H]^{2+}$ = 761.40; $[M+3H]^{3+}$ = 508.00
MP1	1586.06	10.59	$[M+2H]^{2+}$ = 794.40; $[M+3H]^{3+}$ = 530.00
MP2	1543.06	14.00	$[M+2H]^{2+}$ = 772.80; $[M+3H]^{3+}$ = 515.60
MP3	1586.02	10.78	$[M+2H]^{2+}$ = 794.40; $[M+3H]^{3+}$ = 530.00;
MP4	1571.05	2.55	$[M+2H]^{2+}$ = 786.80
MP5	1586.06	21.18	$[M+2H]^{2+}$ = 794.40; $[M+3H]^{3+}$ = 530.00
MP6	1586.06	32.03	$[M+2H]^{2+}$ = 794.40; $[M+3H]^{3+}$ = 530.00
MP7	1571.05	20.37	$[M+2H]^{2+}$ = 786.80
MP8	1571.05	17.82	$[M+2H]^{2+}$ = 786.90
MP9	1544.02	19.17	$[M+2H]^{2+}$ = 773.30; $[M+3H]^{3+}$ = 515.90
MP10	1586.06	13.37	$[M+2H]^{2+}$ = 794.40; $[M+3H]^{3+}$ = 529.90
Cys-MP9-MMP-2-aPD-L1	3692.04	11.08	$[M+3H]^{3+}$ = 1232.60; $[M+4]^{4+}$ = 924.70; $[M+5H]^{5+}$ = 740.00; $[M+6H]^{6+}$ = 616.80
Cys-MP9	1647.03	22.96	$[M+2H]^{2+}$ = 824.90; $[M+3H]^{3+}$ = 550.30
Cys-MMP-2-aPD-L1	2207.06	21.88	$[M+2H]^{2+}$ = 1105.00; $[M+3H]^{3+}$ = 737.10; $[M+4]^{4+}$ = 553.00
FITC-Cys-MP9-MMP-2-aPD-L1	4152.15	2.89	$[M+4H]^{4+}$ = 1039.70; $[M+5H]^{5+}$ = 832.00; $[M+6]^{6+}$ = 693.50; $[M+7H]^{7+}$ = 594.60
FITC-Cys-MP9	2107.14	12.80	$[M+2H]^{2+}$ = 1055.10; $[M+3H]^{3+}$ =703.80; $[M+4H]^{4+}$ = 528.10
FITC-Cys-MMP-2-aPD-L1	2667.17	12.15	$[M+3H]^{3+}$ = 890.60; $[M+4H]^{4+}$ = 668.20
Cys-Lys (N_3)-MP9-MMP2-aPD-L1	3804.11	5.18	$[M+4H]^{4+}$ = 952.00; $[M+5H]^{5+}$ = 761.80; $[M+6H]^{6+}$ = 635.00
Cys-Lys (N_3)-MP9	1759.10	20.69	$[M+2H]^{2+}$ = 879.40; $[M+3H]^{3+}$ = 586.60

Table S2. The conjugation efficiency of peptide-polymer conjugates.

Peptide-polymer conjugate	Conjugation efficiency (%)
PEG-aPDL1	85.15
PEG-MP9	87.75
PEG-MP9-aPDL1	92.02
